# Transcriptome analysis of *Gossypium hirsutum* flower buds infested by cotton boll weevil (*Anthonomus grandis*) larvae

**DOI:** 10.1186/1471-2164-15-854

**Published:** 2014-10-04

**Authors:** Sinara Artico, Marcelo Ribeiro-Alves, Osmundo Brilhante Oliveira-Neto, Leonardo Lima Pepino de Macedo, Sylvia Silveira, Maria Fátima Grossi-de-Sa, Adriana Pinheiro Martinelli, Marcio Alves-Ferreira

**Affiliations:** Department of Genetics, Universidade Federal do Rio de Janeiro - UFRJ Av. Prof. Rodolpho Paulo Rocco, s/n - Prédio do CCS Instituto de Biologia, 2° andar - sala 93, 219410-970 Rio de Janeiro, RJ Brazil; HIV/AIDS Clinical Research Center, Evandro Chagas Clinical Research Institute, Oswaldo Cruz Foundation, Av. Brasil, 4365 - Manguinhos, 21040-360 Rio de Janeiro, RJ Brazil; Embrapa Genetic Resources and Biotechnology, Parque Estação Biológica, PqEB - Av. W5 Norte (final) Caixa Postal 02372, CEP 70770-900 Brasília, DF Brazil; Department of Biochemistry and Molecular Biology, School of Medicine, FACIPLAC, SIGA AE2, Brasília, DF 72460-000 Brazil; Universidade de São Paulo, USP-CENA, Av. Centenário 303, 13416-903 Piracicaba, SP Brazil; University Catholic of Brasilia, Brasília, DF Brazil

**Keywords:** Cotton, Larvae, Transcriptome sequencing, Biotic stress, WRKY FT, Laser microdissection (LMD)

## Abstract

**Background:**

Cotton is a major fibre crop grown worldwide that suffers extensive damage from chewing insects, including the cotton boll weevil larvae (*Anthonomus grandis*). Transcriptome analysis was performed to understand the molecular interactions between *Gossypium hirsutum* L. and cotton boll weevil larvae. The Illumina HiSeq 2000 platform was used to sequence the transcriptome of cotton flower buds infested with boll weevil larvae.

**Results:**

The analysis generated a total of 327,489,418 sequence reads that were aligned to the *G. hirsutum* reference transcriptome. The total number of expressed genes was over 21,697 per sample with an average length of 1,063 bp. The DEGseq analysis identified 443 differentially expressed genes (DEG) in cotton flower buds infected with boll weevil larvae. Among them, 402 (90.7%) were up-regulated, 41 (9.3%) were down-regulated and 432 (97.5%) were identified as orthologues of *A. thaliana* genes using Blastx. Mapman analysis of DEG indicated that many genes were involved in the biotic stress response spanning a range of functions, from a gene encoding a receptor-like kinase to genes involved in triggering defensive responses such as MAPK, transcription factors (WRKY and ERF) and signalling by ethylene (ET) and jasmonic acid (JA) hormones. Furthermore, the spatial expression pattern of 32 of the genes responsive to boll weevil larvae feeding was determined by “*in situ*” qPCR analysis from RNA isolated from two flower structures, the stamen and the carpel, by laser microdissection (LMD).

**Conclusion:**

A large number of cotton transcripts were significantly altered upon infestation by larvae. Among the changes in gene expression, we highlighted the transcription of receptors/sensors that recognise chitin or insect oral secretions; the altered regulation of transcripts encoding enzymes related to kinase cascades, transcription factors, Ca^2+^ influxes, and reactive oxygen species; and the modulation of transcripts encoding enzymes from phytohormone signalling pathways. These data will aid in the selection of target genes to genetically engineer cotton to control the cotton boll weevil.

**Electronic supplementary material:**

The online version of this article (doi:10.1186/1471-2164-15-854) contains supplementary material, which is available to authorized users.

## Background

Cotton is a fibre and oil-yielding crop grown worldwide. Four species of cotton are generally cultivated [1]; however, *Gossypium hirsutum L*. contributes the most to the total lint cotton production worldwide [2]. Cotton productivity is severely affected by both biotic and abiotic stresses [3]. Approximately 1326 species of insects have been reported to attack cotton plants. Among these species, the aphid (*Aphis gossypii G.*), the fall armyworm (*Spodoptera frugiperda*), the budworm (*Heliothis virescens*), the cotton bollworm (*Helicoverpa armigera*) and the cotton boll weevil (*Anthonomus grandis*) are the major pests affecting cotton culture [4]. The cotton boll weevil is undoubtedly the most devastating pest [5, 6]. The adult female feeds, ovoposits, and develops primarily in cotton flower buds and fruits. After hatching, the larvae remain within the reproductive structures and use them as a food sources and a protected habitat to complete their life cycle. The endophytic behaviour of the larvae has made these insects difficult to control with conventional insecticides and other cultural practices [7].

Plants have evolved elaborate defence systems to respond in a rapid and effective way to herbivorous insects. Numerous studies have revealed that, in addition to the constitutive defences comprised of trichomes, thick secondary cell walls, and toxic compounds, plants are equipped with inducible defences that can be grouped into indirect responses, such as the production of volatile odour blends to attract natural enemies of the attacking insect, and direct responses, such as the production of anti-digestive proteins, toxic secondary compounds, and enzymes that affect insect growth and development [8]. These two powerful defence systems evolved by plants during the long arms race with herbivores have enabled plants to survive [9].

An appropriate defence response to a biotic threat requires initial recognition. Herbivores or pathogens are recognised when conserved patterns of molecules, called herbivore- or pathogen-associated molecular patterns, HAMP or PAMP (such as chitin or flagellin) are detected by pattern recognition receptors (PRR) on the surface of the host plant cell, leading to HAMP-triggered immunity (HTI). Damage-associated molecular patterns (DAMP), which are endogenous molecules produced by the plant after infection, are also recognised by PRR to trigger defensive reactions [10, 11]. Following the recognition of an attacker, plants use different signalling cascades to reprogram their phenotype. These include an interconnected network of signal transduction pathways depending mainly on the small regulators jasmonic acid (JA), salicylic acid (SA), and ethylene (ET), concomitantly with Ca^+2^ ion fluxes, mitogen-activated protein kinases (MAPK), transcription factors and reactive oxygen species (ROS) [12].

Crop losses due to insects constitute one of the most significant constraints to the increase of global productivity and food production, estimated at 10-20% for major crops [13]. A better understanding of the diversity of plant responses to insect attacks and, in particular, the induced defences and their regulation, has generated interest in the scientific community to examine alternative strategies to protect plants and crops from insects pests by exploiting the endogenous resistance mechanisms exhibited by plants to most herbivorous insects. Recent transcriptomics studies of plants exposed to herbivory identified a central role for transcripts that can lead to the development of insect-resistant crops [4, 14–18]. High-throughput sequencing of RNA using next-generation sequencing platforms (RNA-Seq) offers a variety of new possibilities such as the transcriptional profiling of organisms lacking sequence information [19], as well as the identification of novel loci, alternative splicing events [20], and sequence variation [21]. Thus, we decided to study the molecular responses of *G. hirsutum* flower buds to infestation by cotton boll weevil (*A. grandis*) larvae using the Illumina HiSeq™ 2000 platform. Furthermore, we combined this approach with laser microdissection (LMD) to isolate RNA from two different regions of tissue damaged by feeding larvae to evaluate the spatial expression pattern of the differentially expressed genes in response to feeding by cotton boll weevil larvae.

## Results

### Analyses of RNA-seq data

To explore the response of *G. hirsutum* to tissue-chewing pests such as cotton boll weevil larvae, the flower bud transcriptome of infested plants was compared with control plants (non-inoculated flower buds). Two biological replicates for each condition were selected for transcriptome analyses with high-throughput parallel sequencing using HiSeq™ 2000, Illumina. We generated 327,489,418 sequence reads, and each sample was represented by at least 74 million reads, a tag density sufficient for quantitative analysis of gene expression (Table 1) [22, 23]. The correlation between the two biological replicates was high (0.99 for infested flower buds and 0.98 for control samples; data not shown).

**Table 1:** **Summary of sequencing data output, statistical analysis of the reads obtained and mapping of the reads onto the cotton (** ***Gossypium hirsutum*** **) transcriptome**

	Total reads^1^	Mapped reads^2^	% of total	OMM^3^	Expressed genes	Yield_Gbases
Sample 3					21,726	8,56
Exon	85,575,328	36,222,891	42.33	18,153,201		
Not mapped	85,575,328	47,769,762	55.82			
Sample 5					21,719	7,43
Exon	74,271,978	32,110,313	43.23	15,672,772		
Not mapped	74,271,978	40,654,742	54.74			
Sample 7					21,697	7,69
Exon	76,936,854	30,018,660	39.02	14,888,504		
Not mapped	76,936,854	45,549,869	59.2			
Sample 8					21,732	9,07
Exon	90,705,258	38,184,759	42.1	18,824,230		
Not mapped	90,705,258	50,726,083	55.92			

^1^Total number of reads mapped onto the G. hirsutum transcriptome.

^2^Percentage of reads mapped onto the G. hirsutum transcriptome.

^3^Number of perfect matches to the reference sequence.

The sequence reads were aligned to the *G. hirsutum* reference transcriptome (cotton EST database) using the BWA package, an efficient engine when searching for perfect matches [24]. Among the total number of reads, 39–43.2% were confined to exons, and 67,538,707 were perfect matches (OMM) to the reference sequence (Table 1). The total number of expressed genes (contigs) was higher than 21,697 per sample (Table 1), and 21,561 expressed genes were common to all samples analysed (data not shown). The average length of contigs generated was 1,063 bp (Additional file 1).

### Overview of the changes in gene expression in response to feeding by cotton boll weevil larvae

The quantitative profiling of the transcriptome using DEGseq (R-bioconductor) analysis identified 443 differentially expressed genes (DEG) in cotton flower buds infested with cotton boll weevil larvae: 402 of them (90.7%) were up-regulated, and 41 (9.3%) were down-regulated compared to the control (adjusted p-value ≤ 0.05, |log FC| ≥ 2.0). Among these DEG, 432 (97.5%) were identified as orthologues of *A. thaliana* genes by Blastx with an e-value of 10^−5^ (Additional file 2). To examine the range of genes involved in the response of cotton flower buds to inoculation with *A. grandis* larvae, the Blast2GO program was also used to confirm the annotation of differentially expressed transcripts (Additional file 2). Blast2GO software returned functions for 87.3% of the differentially expressed genes from species with greater Blast hit distributions that included *Vitis vinifera*, *Glycine max*, *Populus trichocarpa,* and *Threobroma cacao*. Of the DEG, 76% had the same functional annotation between Blastx with *Arabidopsis* and the Blast2GO analysis (Additional file 2).

To determine which genes and pathways were relevant responses to cotton boll weevil larvae feeding, a gene set enrichment analysis (GSEA) approach was used. Based on GSEA, a hypergeometric test (p-values ≤ 0.005) was applied to identify which cellular components (CC), molecular functions (MF) and biological processes (BP) were overrepresented in our list of DEG. GSEA revealed many DEG putatively associated with the plasma membrane and cell wall in the cellular component category (Additional file 3). Most of the genes encoding plasma membrane proteins are receptor-like kinases (RLK), which are known to be involved in the perception of pathogen-derived elicitors. All *RLK* genes are up-regulated in infested plants in comparison to control plants (Additional file 4). Among the genes associated with the cell wall, there are up-regulated transcripts encoding a disease resistance protein (LRR) and cell wall-modifying enzymes, such as pectin methylesterase (*PME41*) and endotransglycosylase/hydrolase proteins (*XTH31*, *XTH32*, *XTH23* and *TCH4*) (Additional file 5). Many of the down-regulated genes encode cytosolic heat shock proteins such as HSP90 and HSP70 (Additional file 5). These molecular chaperones assist in folding newly synthesised proteins and also in several other biological and cellular processes, such as cell growth, development and signal transduction during abiotic and biotic stress [25].

With regard to molecular functions, the DEGs were mainly associated with sequence-specific DNA binding transcription factor activity (Table 2), calmodulin binding and calcium ion binding (Additional file 3).

**Table 2:** **The expression of 88 Gossypium transcription factor (TF) genes in response to cotton boll weevil larvae feeding in flower buds**

Gene family	Contig G. hirsutum	Gene symbol	TAIR code	E-value	Log fold change	Adjusted p-value
Transcription factors						
**WRKY domain transcription factor**						
	**contig8076**	ATWRKY40	AT1g80840	0	4.98	0
	contig1646	ATWRKY40	AT1g80840	0	4.29	0
	contig4254	ATWRKY40	AT1g80840	52.76	2.45	6.94715156272822e-13
	contig313	ATWRKY40	AT1g80840	45.81	2.27	1.59891623026382e-11
	**contig5359**	ATWRKY53	AT4g23810	0	5.37	0
	**contig9783**	ATWRKY70	AT3g56400	0	4.75	1.14115253194648e-13
	contig3807	ATWRKY70	AT3g56400	37.27	2.26	5.24201769462223e-11
	contig3808	ATWRKY70	AT3g56400	34.52	2.24	3.54998511396895e-06
	contig4599	ATWRKY70	AT3g56400	40.27	2.03	6.30528430834368e-05
	**contig4087**	ATWRKY46	AT2g46400	0	4.37	1.76819207423999e-12
	contig4086	ATWRKY46	AT2g46400	37.03	3.5	0
	contig9296	ATWRKY46	AT2g46400	30.99	2.88	8.66053260852238e-14
	**contig1954**	ATWRKY26	AT5g07100	48.26	2.61	4.46309655899313e-14
	**contig4726**	ATWRKY41	AT4g11070	0	4.07	0
	**contig2606**	ATWRKY33	AT2g38470	51.1	3.36	0
	contig2607	ATWRKY33	AT2g38470	53.33	3.14	1.00441679956828e-13
	contig1952	ATWRKY33	AT2g38470	56.58	2.56	3.0123591681817e-14
	contig1953	ATWRKY33	AT2g38470	44.67	2.43	2.45606573427175e-11
	**contig16334**	ATWRKY72	AT5g15130	44.75	2.75	1.43400565479266e-05
	**contig4409**	AtWRKY22	AT4g01250	65.22	2.56	1.56843040084616e-11
**AP2/ERE transcription factor**						
	contig4498	DREB1D	AT5g51990	0	4.88	0
	contig8299	RRTF1	AT4g34410	0	4.85	1.39364892550935e-13
	**contig5510**	ATERF-5	AT5g47230	0	4.7	0
	contig5512	ATERF-5	AT5g47230	0	4.47	0
	contig5102		AT1g63040	0	3.92	0
	contig5511	ATERF-5	AT5g47230	0	3.92	0
	contig18592	CBF4	AT5g51990	0	3.91	8.25514597578304e-13
	contig16446	ATERF-5	AT5g47230	0	3.72	0
	contig18443	DREB26	AT1g21910	0	3.7	0
	contig22401	ATERF-5	AT5g47230	0	3.53	1.03395493612283e-10
	**contig23741**	ATERF98	AT3g23230	61.4	3.45	1.35043960387988e-09
	contig5014	ATERF-5	AT5g47230	44.14	3.17	0
	contig21166		AT1g33760	64	3.11	5.29476229612506e-07
	**contig5559**	RAP2.5	AT3g15210	72.29	3.05	0
	contig13018	ATERF-5	AT5g47230	43.89	2.85	4.47408363363908e-11
	contig23743	ATERF-9	AT5g44210	90.74	2.78	1.53477793062422e-14
	contig11060	ABR1	AT5g64750	55.86	2.69	7.42624465574457e-07
	contig3404	ATERF-9	AT5g44210	47.16	2.63	0
	contig3403	ATERF-9	AT5g44210	49.51	2.26	2.16279246764181e-11
	contig11909		AT5g61890	44.07	2.26	0
	contig2076	ATEBP	AT3g16770	48.21	2.23	5.79122872488872e-11
	contig6750	RAP2.5	AT3g15210	52.26	2.21	1.09859031453532e-10
	contig21935	TINY2	AT5g11590	67.98	2.19	0
**C2H2 zinc-finger**						
	contig21780		AT3g49930	0	3.96	2.21211001647301e-09
	contig3187	ZAT10	AT1g27730	0	3.64	0
	contig567	ZAT10	AT1g27730	0	3.63	5.96999381147476e-12
	contig22821		AT3g49930	42.78	3.39	0
	contig562	STZ	AT1g27730	48.18	3.38	0
	contig3595				3.27	7.24878747340025e-05
	contig563	STZ	AT1g27730	46.85	3.26	0
	contig566	STZ	AT1g27730	64.13	3.2	5.89909045523327e-05
	contig564	STZ	AT1g27730	48.12	2.95	4.46339498962764e-08
	contig569	STZ	AT1g27730	46.67	2.41	9.59875697444564e-12
	contig14282		AT2g28710	53.08	2.34	2.17861060813777e-07
	contig7249		AT3g46070	49.38	2.29	0
**C3H zinc-finger**						
	contig722	ATSZF2	AT2g40140	0	3.54	0
	contig729	CZF1	AT2g40140	53.88	2.95	0
	contig719	CZF1	AT2g40140	62.38	2.79	0
	contig717	CZF1	AT2g40140	52.39	2.59	2.07852782604537e-13
	contig720	ATSZF1	AT3g55980	56.62	3.19	1.77734818473086e-08
	contig728		AT3g55980	50	2.38	0.01
**MYB domain transcription factor**						
	contig11515	ATMYB2	AT2g47190	0	4.31	1.53477793062422e-14
	contig22866	ATMYB73	AT4g37260	0	3.77	0
	contig19903	ATMYB73	AT4g37260	76.92	3.24	1.10170914685033e-09
	contig11954	ATMYB78	AT5g49620	47.7	2.98	0
	contig5383	ATMYB73	AT4g37260	77.88	2.78	0
	contig11235	ATMYB73	AT4g37260	46.84	2.73	1.09623765025238e-08
**GRAS transcription factor**						
	contig3642	SCL5	AT1g50600	62.53	3.24	0
	contig18482	ATGRAS2	AT1g07530	58.33	3.24	3.57762043883002e-10
	contig3507		AT3g46600	49.91	2.73	0
	contig3557	PAT1	AT5g48150	57.06	2.72	0
	contig4834	PAT1	AT5g48150	63.44	2.04	2.21211001647301e-09
**NAC domain transcription factor**						
	contig2875	ANAC002	AT1g01720	59.86	3.04	0
	contig718	ANAC002	AT1g01720	66.98	2.82	0
	contig725	ANAC002	AT1g01720	75.68	2.05	1.37196620183569e-09
	contig4025	NTL9	AT4g35580	72.48	2.45	3.87976777100843e-08
	contig4026	CBNAC	AT4g35580	59.24	2.37	4.87321507933394e-12
	contig14400	ANAC083	AT5g13180	40.79	2.26	1.92653534804078e-07
**bHLH, Basic helix-Loop-helix**						
	contig8902		AT4g20970	0	4.79	0
	contig12411		AT2g22750	0	4.15	1.53477793062422e-14
	contig12410		AT2g22760	0	3.53	8.73481683691145e-06
	contig21436		AT3g07340	41.01	3.15	4.49986558503901e-05
	contig24140		AT5g57150	48	2.83	3.38680046143333e-13
	contig23410		AT3g07340	52.34	2.69	8.59443189061066e-06
	contig24822	BANQUO 1	AT5g39860	67.95	2.65	2.26903319960744e-10
	contig10541		AT1g10120	70.41	2.22	9.34996779453853e-11
	contig5829		AT4g20970	38.85	2.22	0
**HSF, Heat-shock transcription factor**						
	contig1779	ATHSFA2	AT2g26150	50.39	−3.29	0

Genes in bold were tested by qPCR.

**Figure 1:**
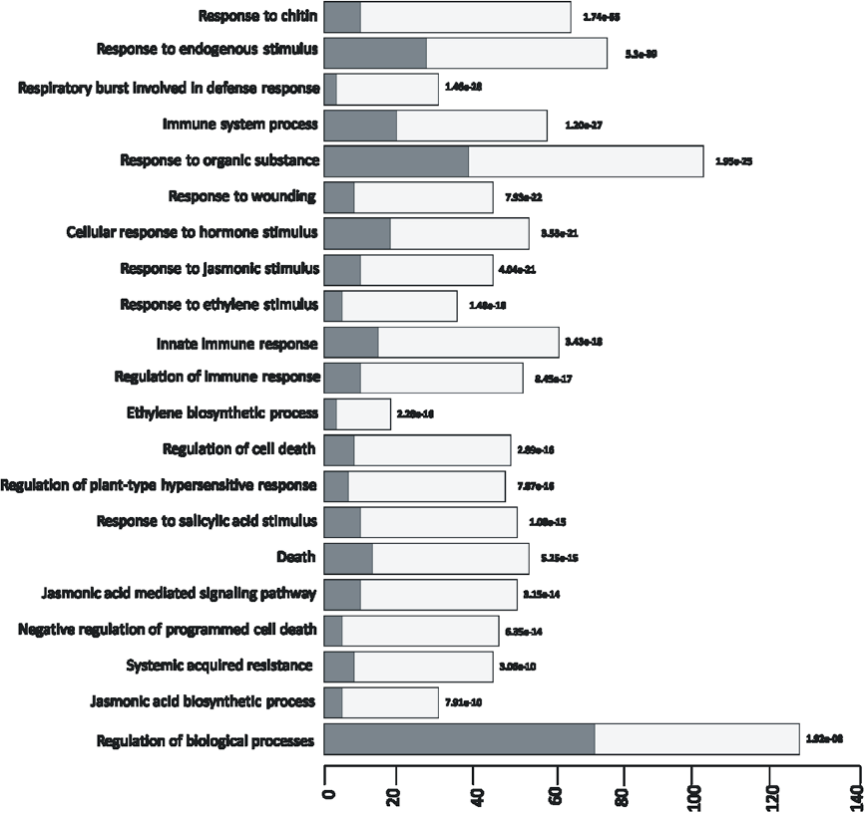
**Distribution of differentially expressed genes (DEGs) (x-axis) into Gene Ontology (GO) categories (biological process) (y-axis) according to Gene Set Enrichment Analysis (GSEA).** Only biological processes (BPs) discussed in the results are presented here. A complete list of BPs can be found in Additional file 6.

Furthermore, GSEA revealed significant biological processes altered in plants upon infection with cotton boll weevil larvae. We found that biological processes related to hormone biosynthesis and signalling, the response to organic substances, the regulation of biological processes, the detection of biotic stimuli, systemic acquired resistance, the respiratory burst involved in the defence response and innate immune responses were overrepresented by GSEA (Figure 1). Moreover, biological processes associated with defence against insects, such as the response to chitin, the regulation of the plant-type hypersensitive response (HR), the regulation of programmed cell death and death were also represented (Figure 1). One hundred thirty-four transcripts were found in the “response to chitin” category (GO:0010200), and 36 of them were also annotated with the gene ontology (GO) term “death” (GO:0016265) (Table 3). Of these, many transcripts encoded proteins involved in signal transduction such as receptor-like kinases, mitogen-activated protein kinases, calcium ion binding proteins and calmodulin-like proteins. In addition to genes associated with signal transduction, genes associated with hormone biosynthesis and the 26S proteasome pathway were annotated with the GO term “response to chitin and death” (Additional file 6).

**Figure 2:**
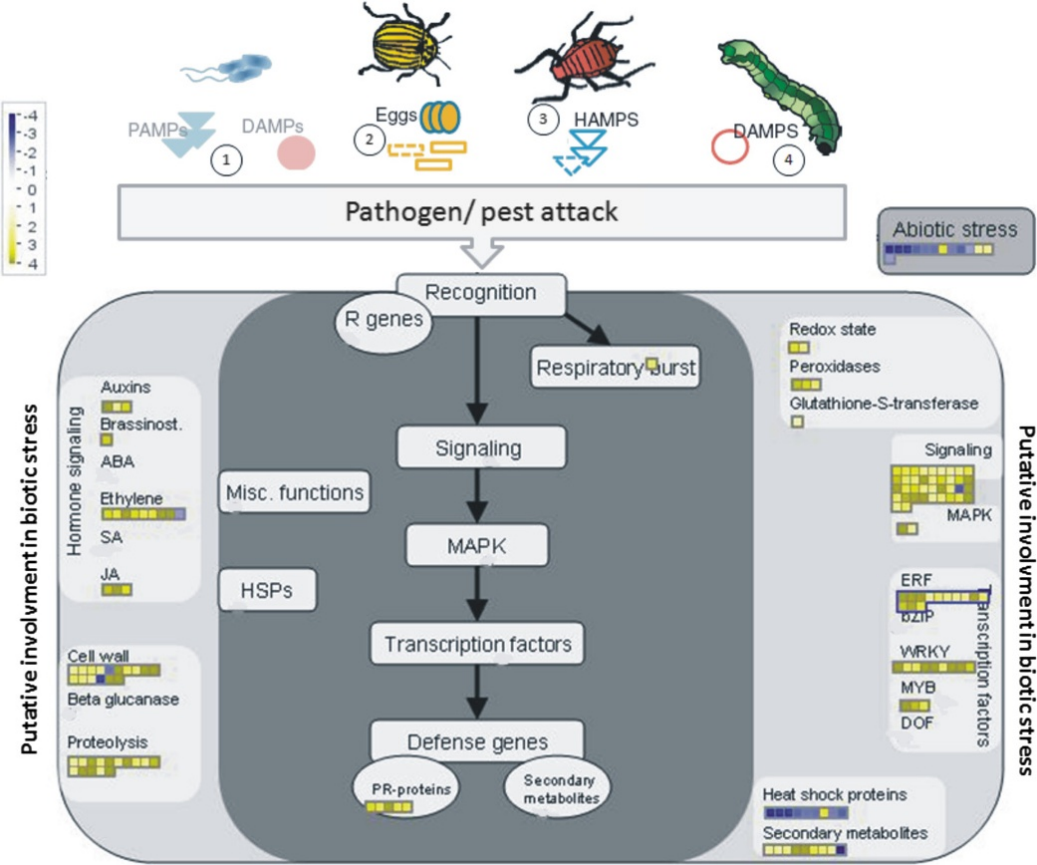
**Representative overview of DEG involved in biotic stress response in** ***Gossypium hirsutum*** **48 h after infection with cotton boll weevil larvae.** Log2-fold change of gene expression (cotton boll weevil compared to mock-inoculated control) was analysed by MapMan software. Yellow squares represent up-regulated genes and blue squares represent down-regulated genes. The colour saturation indicates log fold change > 4 and < 4. The figure shows that the molecular recognition of pathogens and herbivores by plants to trigger a defence response requires initial recognition including the following: 1. Microbe-, pathogen- and damage-associated molecular patterns (MAMP, PAMP and DAMP) are recognised by pattern recognition receptors (PRR) and lead to PAMP-triggered immunity (PTI). 2. Oviposition-associated compounds are recognised by unknown receptors and trigger defensive responses. 3. Putative herbivore-associated molecular patterns (HAMP) are recognised by receptors and lead to herbivore-triggered immunity (HTI). 4. Wounding by herbivores leads to the release of DAMP and to wound-induced resistance (WIR).

The DEGs were mapped using MapMan to generate a representative overview of the pathways affected (Figure 2). This analysis indicated the involvement of several genes in the biotic stress response including receptors recognising microbe-, pathogen-, herbivore- and damage-associated molecular patterns (MAMP, PAMP, HAMP and DAMP) as well as genes involved in triggering defensive responses such as transcription factors and the ET and JA signalling pathways (Figure 2).

**Table 3:** **Subset of differentially expressed genes (DEG) in cotton flower buds in response to feeding by cotton boll weevil larvae**

Contigs cotton	Gene symbol	TAIR annotation	SubjectID	logFold change	Adjusted P-value
**GO:0010200 - Response to chitin**					
contig7179*		Protein kinase family protein with leucine-rich repeat domain	AT5g25930	2.02	5.33E-09
contig14965	Kin3	Encodes a putative serine/threonine-specific protein kinase	AT2g17220	2.03	1.20E-08
contig21694		Protein kinase superfamily	AT1g18390	2.65	5.45E-10
contig2968	CPK28	Member of Calcium Dependent Protein Kinase	AT5g66210	2.47	0.01
**contig17371**	CCR4	Serine/threonine-protein kinase-like domain	AT5g47850	3.51	0
contig2100	MAPK3	Encodes a mitogen-activated kinase	AT5g57510	4.27	8.61E-07
**contig2102**	MAPK3	Encodes a mitogen-activated kinase	AT3g45640	2.17	2.57E-10
**contig14909**	MKK9	Member of MAP Kinase Kinase family	AT1g73500	2.22	1.58E-10
contig7892	MAPKKK14	Member of MEKK subfamily	AT2g30040	2.96	4.79E-11
contig4523		ATPase E1-E2 type family protein/haloacid dehalogenase-like hydrolase family protein	AT3g63380	3.3	0
contig11388		ATPase E1-E2 type family protein/haloacid dehalogenase-like hydrolase family protein	AT3g63380	2.2	7.24E-11
contig18754		ATPase E1-E2 type family protein/haloacid dehalogenase-like hydrolase family protein	AT3g63380	2.15	3.53E-09
contig7822	CML38	Calmodulin-like 38 (CML38)	AT1g76650	4.87	1.56E-06
**contig7823**	CML38	Calmodulin-like 38 (CML38)	AT1g76650	3.32	0
**contig535**	CML24	Encodes a protein with 40% similarity to calmodulin	AT5g37770	3.48	0
contig2687		C2 calcium-dependent membrane targeting	AT2g25460	4.02	0
contig12364		Calcium-binding EF-hand family protein	AT4g27280	4.1	0
contig2688		C2 calcium-dependent membrane targeting	AT2g25460	3.89	1.72E-11
contig14266*	ACA2	Encodes a calmodulin-regulated Ca(2+)-pump located in the endoplasmic reticulum	AT4g37640	2.17	2.26E-10
contig2595*	ACA2	Encodes a calmodulin-regulated Ca(2+)-pump located in the endoplasmic reticulum	AT4g37640	2.15	7.16E-10
contig8294*	EDA39	Encodes a calmodulin-binding protein involved in stomatal movement	AT4g33050	2.61	3.93E-12
contig1798	PUMP5	Encodes one of the mitochondrial dicarboxylate carriers (DIC)	AT2g22500	6.13	0
contig9093*		Encodes a cell wall bound peroxidase	AT5g64120	3.76	4.46E-14
contig9094*		Encodes a cell wall bound peroxidase	AT5g64120	3.42	0
contig8534*	DMR6	Encodes a putative 2OG-Fe(II) oxygenase	AT5g24530	2.25	1.53E-06
contig1723*		Protein phosphatase 2C family protein	AT4g33920	3.07	7.56E-13
contig1722*		Protein phosphatase 2C family protein	AT4g33920	2.7	1.53E-14
contig1720*		Protein phosphatase 2C family protein	AT4g33920	2.32	4.51E-07
contig8378		Acyl-CoA N-acyltransferases (NAT) superfamily protein	AT2g32030	4.48	0
contig24379*	PLA/PLA2A	Encodes a lipid acyl hydrolase	AT2g26560	3.15	0
contig11992		Alpha/beta-Hydrolases superfamily protein	AT3g19970	3.13	1.89E-10
contig1674	TCH4	Encodes a cell wall-modifying enzyme	AT5g57560	5.84	9.62E-12
contig2539	TCH4	Encodes a cell wall-modifying enzyme	AT5g57560	5.57	0
contig1676	TCH4	Encodes a cell wall-modifying enzyme	AT5g57560	4.66	0
contig2101	TCH4	Encodes a cell wall-modifying enzyme	AT5g57560	4.63	0
contig2538	TCH4	Encodes a cell wall-modifying enzyme	AT5g57560	3.72	8.85E-13
contig2536	TCH4	Encodes a cell wall-modifying enzyme	AT5g57560	3.22	1.27E-13
contig4524	TCH4	Encodes a cell wall-modifying enzyme	AT5g57560	5.53	0
contig9042	RRTF1	Member of the ERF (ethylene response factor) of ERF/AP2 transcription factor family	AT4g34410	37.97	0
contig8299	RRTF1	Member of the ERF (ethylene response factor) of ERF/AP2 transcription factor family	AT4g34410	4.85	1.39E-13
contig8860		Member of the DREB subfamily A-5 of ERF/AP2 transcription factor family	AT1g19210	9.53	0
contig23571		Member of the DREB subfamily A-5 of ERF/AP2 transcription factor family	AT1g19210	7.29	0
contig24042		Member of the DREB subfamily A-5 of ERF/AP2 transcription factor family	AT1g19210	6.35	0
contig13134		Member of the DREB subfamily A-5 of ERF/AP2 transcription factor family	AT5g51190	5.25	0
contig19639		Member of the DREB subfamily A-5 of ERF/AP2 transcription factor family	AT1g19210	5.01	0
**contig23741**	ERF98	Member of the ERF (ethylene response factor)	AT3g23230	3.45	1.35E-09
**contig5510**	ERF-5	Member of the ERF (ethylene response factor)	AT5g47230	4.7	0
contig5512	ERF-5	Member of the ERF (ethylene response factor)	AT5g47230	4.47	0
contig5511	ERF-5	Member of the ERF (ethylene response factor)	AT5g47230	3.92	0
contig16446	ERF-5	Member of the ERF (ethylene response factor)	AT5g47230	3.72	0
contig22401	ERF-5	Member of the ERF (ethylene response factor)	AT5g47230	3.53	1.03E-10
contig5014	ERF-5	Member of the ERF (ethylene response factor)	AT5g47230	3.17	0
contig13018	ERF-5	Member of the ERF (ethylene response factor)	AT5g47230	2.85	4.47E-11
**contig5559**	ERF-4	Member of the ERF (ethylene response factor)	AT3g15210	3.05	0
contig6750	ERF-4	Member of the ERF (ethylene response factor)	AT3g15210	2.21	1.10E-10
contig8076*	GhWRKY40-like4	Pathogen-induced transcription factor	AT1g80840	4.98	0
contig1646*	GhWRKY40-like3	Pathogen-induced transcription factor	AT1g80840	4.29	0
contig4254*	GhWRKY40-like1	Pathogen-induced transcription factor	AT1g80840	2.45	6.95E-13
contig313*	GhWRKY40-like2	Pathogen-induced transcription factor	AT1g80840	2.27	1.60E-11
contig5359*	GhWRKY64-like1	Member of WRKY Transcription Factor; Group III	AT4g23810	5.37	0
contig9783*	GhWRKY70-like1	Member of WRKY Transcription Factor; Group III	AT3g56400	4.75	1.14E-13
contig3807*	GhWRKY70-like3	Member of WRKY Transcription Factor; Group III	AT3g56400	2.26	5.24E-11
contig3808*	GhWRKY70-like4	Member of WRKY Transcription Factor; Group III	AT3g56400	2.24	3.55E-06
contig4599*	GhWRKY70-like2	Member of WRKY Transcription Factor; Group III	AT3g56400	2.03	6.31E-05
**contig4087**	GhWRKY46-like1	Member of WRKY Transcription Factor; Group III	AT2g46400	4.37	1.77E-12
contig9296	GhWRKY46-like3	Member of WRKY Transcription Factor; Group III	AT2g46400	2.88	8.66E-14
contig4086	GhWRKY46-like2	Member of WRKY Transcription Factor; Group III	AT2g46400	3.5	0
contig2606*	GhWRKY33-like1	Member of the plant WRKY transcription factor family	AT2g38470	3.61	0
contig2605*	GhWRKY33-like2	Member of the plant WRKY transcription factor family	AT2g38470	3.36	0
contig1952	GhWRKY33-like3	Member of the plant WRKY transcription factor family	AT2g38470	2.56	3.01E-14
contig1953*	GhWRKY33-like4	Member of the plant WRKY transcription factor family	AT2g38470	2.43	2.46E-11
**contig4409**	GhWRKY22-like1	member of WRKY Transcription Factor; Group II-e	AT4g01250	2.56	1.57E-11
contig565	STZ	Related to Cys2/His2-type zinc-finger proteins	AT3g63380	5.4	0
contig6454	STZ	Related to Cys2/His2-type zinc-finger proteins	AT1g27730	5.22	0
contig561	STZ	Related to Cys2/His2-type zinc-finger proteins	AT1g27730	5.07	0
contig3187	STZ	RING-H2 protein induced after exposure to chitin	AT1g27730	3.64	0
contig1541*	ATL2	RING-H2 protein induced after exposure to chitin	AT3g16720	3.77	0
contig1540*	ATL2	RING-H2 protein induced after exposure to chitin	AT3g16720	3.71	0
contig3187	STZ	Related to Cys2/His2-type zinc-finger proteins found in higher plants	AT1g27730	3.64	0
contig567	STZ	Related to Cys2/His2-type zinc-finger proteins found in higher plants	AT1g27730	3.63	5.97E-12
contig563	STZ	Related to Cys2/His2-type zinc-finger proteins found in higher plants	AT1g27730	3.26	0
contig566	STZ	Related to Cys2/His2-type zinc-finger proteins found in higher plants	AT5g57560	3.2	5.90E-05
contig569	STZ	Related to Cys2/His2-type zinc-finger proteins found in higher plants	AT1g27730	2.41	9.60E-12
contig562	STZ	Related to Cys2/His2-type zinc-finger proteins found in higher plants	AT1g27730	3.38	0
contig5663		Related to Cys2/His2-type zinc-finger proteins found in higher plants	AT1g27730	3.63	0
contig564	ATSZF2	Domain Zinc finger, C3H-type	AT2g40140	2.95	4.46E-08
contig722*	ATSZF2	Domain Zinc finger, C3H-type	AT2g40140	3.54	0
contig729*	ATSZF2	Zinc finger, C3H-type	AT2g40140	2.95	0
contig719*	ATSZF2	Zinc finger, C3H-type	AT2g40140	2.79	0
contig717*	ATSZF2	Zinc finger, C3H-type	AT2g40140	2.59	2.08E-13
contig720	ATSZF1	Salt-inducible zinc finger 1 (SZF1)	AT3g55980	3.19	1.78E-08
contig728	ATSZF1	Salt-inducible zinc finger 1 (SZF1)	AT3g55980	2.38	0.01
contig5654	BRH1	Encodes a novel ring finger protein	AT3g61460	3.61	0
contig22866	ATMYB73	Member of the R2R3 factor gene family	AT3g16720	3.77	0
contig19903	ATMYB73	Member of the R2R3 factor gene family	AT4g37260	3.24	1.10E-09
contig5383	ATMYB73	Member of the R2R3 factor gene family	AT4g37260	2.78	0
contig11235	ATMYB73	Member of the R2R3 factor gene family	AT4g37260	2.73	1.10E-08
contig3507	GRAS	GRAS family transcription factor	AT3g46600	2.73	0
contig2875	ANAC002	Belongs to a large family of putative transcriptional activators with NAC domain	AT1g01720	3.04	0
contig718	ANAC002	Belongs to a large family of putative transcriptional activators with NAC domain	AT1g01720	2.82	0
contig725	ANAC002	Belongs to a large family of putative transcriptional activators with NAC domain	AT1g01720	2.05	1.37E-09
contig10852	ATL6	Encodes a putative RING-H2 zinc finger protein ATL6 (ATL6)	AT3g05200	2.27	1.63E-10
contig7248	RHL41	Encodes a zinc finger protein	AT5g59820	2.66	5.29E-06
contig1779	ATHSFA2	Member of Heat Stress Transcription Factor (Hsf) family	AT2g26150	−3.29	0
contig18462*	BETA	Encodes a beta carbonic anhydrase likely to be localized in the cytoplasm	AT5g14740	2.69	0
contig4843		Encodes an ABA- and drought-induced RING-DUF1117 gene	AT5g59550	2.09	1.56E-07
**contig17118**	AOS	Member of the cytochrome p450 CYP74 gene family that functions as an allene oxide synthase	AT5g42650	4.5	2.74E-09
**contig3758**	ACS	Encodes a a member of the 1-aminocyclopropane-1-carboxylate (ACC) synthase	AT4g11280	3.48	0
contig12553*	ACO	Encodes 1-aminocyclopropane-1-carboxylate oxidase	AT1g05010	3.1	0
contig1208*	ACO	Encodes 1-aminocyclopropane-1-carboxylate oxidase	AT1g05010	2.16	1.48E-10
contig1206*	ACO	Encodes 1-aminocyclopropane-1-carboxylate oxidase	AT1g05010	2.09	7.20E-10
contig1960	LOX	PLAT/LH2 domain-containing lipoxygenase family protein	AT1g72520	3.73	0
**contig9217**	CYP707A3	Encodes a protein with ABA 8′-hydroxylase activity	AT5g45340	3.19	0
contig4960*	JAZ1	JAZ1 is a nuclear-localized protein involved in jasmonate signaling	AT1g19180	2.26	5.01E-11
contig4959*	JAZ1	JAZ1 is a nuclear-localized protein involved in jasmonate signaling	AT1g19180	2.19	2.02E-09
contig15546	JAZ7	Jasmonate-zim-domain protein 7 (JAZ7)	AT2g34600	2.03	0
contig5978	ATCHITIV	Encodes an EP3 chitinase	AT3g54420	3.08	1.53E-14
contig21823	ATCHITIV	Encodes an EP3 chitinase	AT3g54420	2.57	2.26E-10
contig6127	AtCAF1a	Encodes one of the homologs of the yeast CCR4-associated factor 1	AT3g44260	2.94	0
contig1389	AT-SYR1	Encodes a syntaxin localized at the plasma membrane	AT3g11820	2.82	5.38E-12
contig1388	AT-SYR1	Encodes a syntaxin localized at the plasma membrane	AT3g11820	2.41	2.02E-12
contig5585	ATHSPRO2	Ortholog of sugar beet HS1 PRO-1 2 (HSPRO2)	AT2g40000	2.69	0
contig3792	ATHSPRO2	Ortholog of sugar beet HS1 PRO-1 2 (HSPRO2)	AT2g40000	2.16	6.93E-05
contig2278		Encodes an ABA- and drought-induced RING-DUF1117 gene	AT5g59550	2.61	1.53E-14
contig4842		Encodes an ABA- and drought-induced RING-DUF1117 gene	AT5g59550	2.41	8.61E-06
contig4022	ATNHL10	Encodes a protein whose sequence is similar to tobacco hairpin-induced gene (HIN1)	AT2g38470	2.56	8.82E-06
contig4024	ATNHL10	Encodes a protein whose sequence is similar to tobacco hairpin-induced gene (HIN1)	AT2g35980	2.26	1.06E-09
contig22620		Hs1pro-1 protein	AT3g55840	2.53	1.76E-09
contig3055	(AT)SRC2	Unknown protein	AT1g09070	2.38	6.49E-12
contig6505		ARM repeat superfamily protein	AT4g27280	4.04	0
contig17616*	PUB23	Encodes a cytoplasmically localized U-box domain containing E3 ubiquitin ligase	AT2g35930	4.21	4.10E-12
contig2098	ATPUB29	E3 ubiquitin ligase activity	AT3g18710	3.95	0
**contig6504**	ATCMPG1	Ubiquitin-protein ligase activity	AT1g66160	3.9	0
contig8776		Ubiquitin-protein ligase activity	AT5g37490	2.31	2.24E-07
***GO:0016265 - Death***					
contig10876	RLK1	Encodes a receptor-like protein kinase	AT5g60900	2.56	5.07E-05
contig15035	RLK1	Encodes a receptor-like protein kinase	AT5g60900	2.28	8.92E-06
**contig6958**	RIPK	Encodes a receptor-like cytoplasmic kinase	AT2g05940	2.13	1.14E-09
**contig5615**	EVR	Encodes a putative leucine rich repeat transmembrane protein kinase	AT2g31880	2.21	7.45E-09
contig12017	CCR3	CRINKLY4 related 3 (CCR3); kinase activity	AT3g55950	2.24	3.33E-07
contig7179	NA	Protein kinase family protein with leucine-rich repeat domain	AT5g25930	2.02	5.33E-09
contig10122	ATPP2-A1	Encodes a phloem lectin	AT4g19840	2.79	0
**contig7573**	GRX480	Encodes GRX480, a member of the glutaredoxin family that regulates protein redox state	AT1g28480	2.35	2.97E-12
**contig8179**	ATRBOHD	NADPH/respiratory burst oxidase protein D (RbohD)	AT5g47910	2.64	1.53E-14
contig9014	ATRBOHD	NADPH/respiratory burst oxidase protein D (RbohD)	AT5g47910	2.48	7.49E-11
contig9015	ATRBOHD	NADPH/respiratory burst oxidase protein D (RbohD)	AT5g47910	2.65	1.57E-09
contig4745	ATMC1	Metacaspase AtMCP1b	AT1g02170	2.24	1.02E-09
contig5579	ATGATL1	The PARVUS/GLZ1 gene encodes a putative family 8 glycosyl transferase	AT1g19300	2.66	0
contig2607	ATWRKY33	Member of the plant WRKY transcription factor family	AT2g38470	3.14	1.00E-13
contig2076	ATEBP	Encodes a member of the ERF (ethylene response factor)	AT3g16770	2.23	5.79E-11
contig14400	ANAC083	Encodes a NAC domain transcription factor	AT5g13180	2.26	1.93E-07
contig12668	JMT	Encodes a S-adenosyl-L-methionine:jasmonic acid carboxyl methyltransferase	AT1g19640	3.4	0
contig13244	AtLEA5	Encodes AtLEA5, also known as Senescence-associated gene 21 (SAG21). Has a role on oxidative stress tolerance	AT4g02380	2.34	7.06E-12
contig4041	AtLEA5	Encodes AtLEA5, also known as Senescence-associated gene 21 (SAG21). Has a role on oxidative stress tolerance	AT4g02380	2.76	0
contig4042	AtLEA5	Encodes AtLEA5, also known as Senescence-associated gene 21 (SAG21). Has a role on oxidative stress tolerance	AT4g02380	2.37	2.58E-09
contig4022	ATNHL10	Encodes a protein non-race specific disease resistance gene (NDR1)	AT2g35980	2.56	8.82E-06
contig4024	ATNHL10	Encodes a protein non-race specific disease resistance gene (NDR1)	AT2g35980	2.26	1.06E-09
contig3055	(AT)SRC2	SRC2 specifically binds the peptide PIEPPPHH	AT1g09070	2.38	6.49E-12
contig1388	AT-SYR1	Encodes a syntaxin	AT3g11820	2.41	2.02E-12
contig1389	AT-SYR1	Encodes a syntaxin	AT3g11820	2.82	5.38E-12
contig21823	ATCHITIV	Encodes an EP3 chitinase	AT3g54420	2.57	2.26E-10
contig5978	ATCHITIV	Encodes an EP3 chitinase	AT3g54420	3.08	1.53E-14

Only DEG with the GO term for the biological process “response to chitin” (GO:0010200) and/or “death” (GO:0016265) are shown. The complete list of DEG is shown in Additional file 2.

*Genes present in both biological processes: response to chitin and death.

Genes in bold were tested by qPCR.

### Main processes or pathways affected by the response to infection with cotton boll weevil larvae

Several subsets of genes were identified, including protein kinases, Ca^2+^-binding sensor proteins (CaM), genes responsive to oxidative stress, transcription factors, cell-wall modification genes and phytohormone-responsive genes.

### Genes associated with signal transduction: kinases, Ca^2+^-binding proteins and protein degradation

An appropriate defence response to a biotic threat initially requires recognition of the threat. Herbivore-associated molecular patterns such as chitin are detected by pattern recognition receptors (PRR) on the surface of the host plant cell, leading to HAMP-triggered immunity. Well-characterised plant PRR comprises leucine-rich repeat (LRR) receptor-like kinases (RLK). Members of this family of proteins have a common structure formed by an extracellular ligand-binding domain and a cytoplasmic kinase domain [26]. Eleven RLK/LRR are differentially expressed (up-regulated) and annotated with the GO term “response to chitin” and/or “death” (Table 3), including receptor-like cytoplasmic kinase (*RIPK*), receptor-like kinase localised on the plasma membrane (*RLK1*), serine/threonine-protein kinase-like domain (*CCR4* and *CCR3*) and putative leucine rich repeat transmembrane protein kinase (*EVR*). Recognition of conserved PAMP by PRR triggers intracellular signalling via mitogen-activated protein kinase (*MAPK*, *MAPKK*, *MAPKKK*) cascades followed by the activation of WRKY transcription factors [27]. In our analysis, cotton boll weevil larvae affected the expression pattern of several mitogen-activated protein kinase genes. For instance, two mitogen activated protein kinases (MAPK) that encode MAPK3 in *A. thaliana* were transcriptionally up-regulated (contig2100 and contig2102). Similarly, a member of the MAP Kinase Kinase family (MKK9, contig14909) and a protein kinase kinase kinase (MAPKKK14, contig7892) that play a role in leaf senescence during pathogenesis by *Alternaria blight* in *A. thaliana* were also transcriptionally up-regulated [28]. The activation of MAPK through wounding is dependent on the direct binding of calmodulins (CaM) in a Ca^2+^ dependent manner. Mechanical wounding and insect attack trigger a transient increase of cytosolic Ca^2+^ levels within minutes. This Ca^2+^ oscillation acts as a mediator for stimulus responses to several Ca^2+^-binding proteins, such as calmodulins (CaM), calcineurin B-like proteins, and calcium-dependent protein kinases (CDPK) [29]. In our analysis, nine transcripts coding for calmodulin-like proteins, including *EDA39*, *CML38*, *CML24* and *ACA2*, were present in the DEG related to chitin response. In Arabidopsis, these genes are involved in plant innate immunity signalling and function as sensors of the signal generated by Ca^2+^ influx into the cytosol (Table 3) [30].

Targeted protein degradation via the ubiquitin/26S-proteasome pathway is another important regulatory process. Among the thirteen *G. hirsutum* genes annotated to be involved in this process, four were also related to the chitin and/or death GO (Table 3).

### Cotton boll weevil-induced transcription factor (TF) genes

Transcription factors serve as important regulators of biotic and abiotic stress responses by turning on or off the immune system during plant defence. Our results suggest that the gene expression reprogramming in response to the boll weevil larvae feeding depends on many transcription factors (Table 2). As many as 88 TFs, which are 20% of the DEGs, were differentially expressed after cotton boll weevil infection, with 87 up-regulated and only one down-regulated (*HSF*, Heat-Shock transcription factor). Among the TFs up-regulated by cotton boll weevil feeding, there are 23 ethylene response factors (AP2/ERE), 21 WRKY, 12 C2H2 zinc finger, nine helix–loop–helix (bHLH), six C3H zinc finger, six MYB, six NAC and five GRAS (Table 2). Members of the AP2/ERE and WRKY families were also overrepresented in the study reported by Libault et al. [31], in which the expression patterns of *Arabidopsis* transcription factors were analysed in response to purified chitooctaose. Similarly, Wei et al. [14] observed that among the differentially expressed genes in *Arabidopsis* leaves in response to diamond back moth feeding, there were 18 ethylene response factors (ERFs) and 10 WRKY. The ERF subfamily belongs to the APETALA2 (AP2)/ethylene-responsive-element-binding protein (EREBP) family, an FT family exclusive to plants. WRKY transcription factors in plants are one of the largest families of zinc finger transcription factors and modulate development as well as responses to abiotic stresses, wounding, pathogens, and herbivore attack [32]. WRKY TFs can act as both positive and negative regulators of plant defence pathways. The mechanisms activating WRKY TF might involve the MAP kinase cascades and/or calcium signalling [32–34].

**Figure 3:**
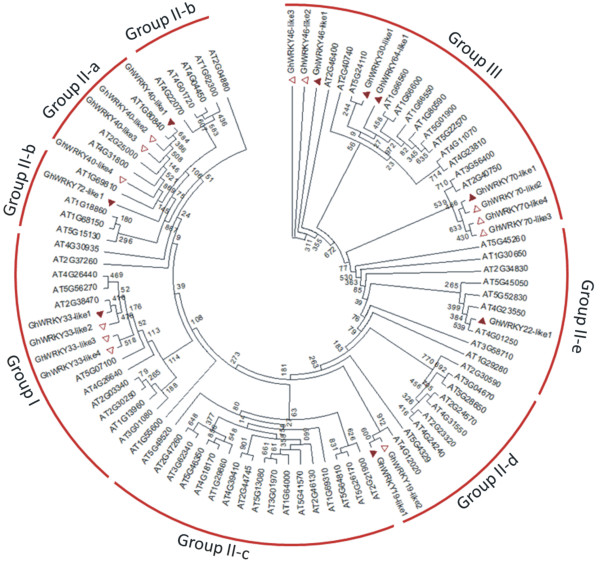
**Phylogenetic tree of WRKY domains between cotton and** ***Arabidopsis*** **.** The amino acid sequences of the WRKY domain of cotton and *Arabidopsis* were aligned with MUSCLE, and the phylogenetic tree was constructed using the JTT model with an estimated γ-distribution parameter (G). The maximum-likelihood analyses were performed with the program PhyML version 3.0, and assessment of node confidence was performed using 1,000 bootstrap replicates. The members of group I, II (a-e) and III are labelled according to the classifications of AtWRKY domains by Eulgem et al. [33]. The triangles indicate cotton WRKY genes, and filled triangles represent the genes analysed by qPCR. Contig9787 was named *GhWRKY70-like1*; was named contig5359 *GhWRKY64-like1*; and contig16334 was named *GhWRKY72-like1* after calculating the p-distance to determine the closest relationship with *Arabidopsis* members.

In the present study, 21 WRKYs were up-regulated (Table 2). Considering the large number of genes in this family that were up-regulated in our experiment and their importance in the response to pathogen and herbivore attack, we investigated this TF family in more detail. A phylogenetic analysis including *WRKY* genes from cotton and *Arabidopsis* was performed (Figure 3 and Additional file 7). The phylogenetic analysis classified the cotton genes in the WRKY clades and identified several putative cotton homologues to *Arabidopsis* WRKYs that have been previously characterised. Among the genes with an altered response to cotton boll weevil larvae feeding, we identified nine WRKY genes in cotton with putative homologues in *Arabidopsis* that belong to group III: WRKY46, WRKY30, WRKY64 and WRKY70 (Figure 3 and Additional file 7) [33, 34]. In addition, *GhWRKY40-like1* (AT1G80840), *GhWRKY33-like1* (AT2G38470) and *GhWRKY22-like1* (AT4G01250) genes belong to group IIa, I, and IIe, respectively (Figure 3 and Additional file 7). Other zinc finger-containing transcription factors from the C2H2 and C3H families were also up-regulated during boll weevil larvae feeding. Members of these families are also up-regulated by aphid and *Pseudomonas syringae* attack in *Arabidopsis* [35] (Table 2). MYB proteins are central regulators of development, metabolism, and the response to abiotic and biotic stresses [36]. In our analysis, we found six up-regulated genes coding for R2R3-MYB TFs (Table 2). Defence responses regulated by MYB transcription factors promote HR-related cell death and resistance against bacterial and necrotrophic pathogens. MYB transcription factors also play roles in the defence response against insects. Small Cabbage White caterpillars (*Pieris rapae*) induce local expression of *AtMYB102* [37]. NAC transcription factors have been shown to regulate plant development, phytohormone signalling and abiotic stress responses [38]. Six NAC genes were differentially expressed in response to cotton boll weevil larvae feeding (Table 2). Some NAC genes are up-regulated in response to feeding by diamond back moths, wounding, and bacterial infection [39, 40]. bHLH transcription factors regulate plant cell and tissue development as well as phytohormone signalling. A limited number of bHLH transcription factors characterised to date have been found to be involved in the defence against pathogens or pests. Nine bHLH TFs were consistently up-regulated in this study. Other differentially expressed (up-regulated) transcription factors belonging to the GRAS family play diverse roles in root and shoot development, gibberellic acid (GA) signalling, phytochrome A signal transduction and the chitin induced defence response [41, 42]. We focused on three members of the AP2/ERE family and nine members of the WRKY family to further characterise the response to cotton boll weevil feeding by qPCR analysis (Table 2).

### Oxidative stress-related genes affected by cotton boll weevil feeding

Oxidative radicals play an important role in plants during various stresses, including biotic stress such as insect infestation [43]. Among the genes up-regulated by oxidative stress, a transcript encoding the respiratory burst oxidative homolog D (*RbohD*) protein, a key enzyme involved in the reduction of reactive oxygen species (ROS) during the defence response, was identified. In addition, *GRX480*, a member of the glutaredoxin family that catalyses thiol disulphide reduction and might be a candidate controlling the redox state of regulatory proteins, was also up-regulated. *GRX480* might represent a potential regulatory component of SA/JA antagonism [44].

### Phytohormone-related genes induced by the boll weevil larvae feeding

Phytohormones are crucial in the stimulation of defence to insect herbivory. Several signalling pathways, including JA, SA, and ET, are believed to orchestrate the induction of insect defences [10]. Genes associated with the biosynthesis, signalling and response to stimuli by JA, SA, ABA (abscisic acid) and ET phytohormones were significantly overrepresented in the flower bud transcriptome affected by the boll weevil based on hypergeometric distribution analysis (p-values ≤ 0.005) (Figure 1). For instance, the genes associated with jasmonate biosynthesis are well represented among the genes up-regulated by cotton boll weevil feeding, including lipoxygenase (*LOX*), allene oxide synthesis (*AOS*), and jasmonic acid carboxyl methyltransferase (*JMT*). We identified genes associated with salicylic acid signalling, a widely known phytohormone involved in the induction of pathogen resistance (PR) genes as well as in the establishment of long-term immunity via the SAR pathways [45] (Figure 1). In addition, other genes up-regulated by boll weevil feeding are involved in ABA catabolism and ET biosynthesis. These include the gene encoding the ABA 8′-hydroxylase (*CYP707A3*), which catalyses the first step in the oxidative inactivation of ABA [46]. The genes involved in ethylene biosynthesis *ACS* (1-aminocyclopropane-1-carboxylate synthase) and *ACO* (1-aminocyclopropane-1-carboxylate oxidase) were also up-regulated after herbivory (Table 3).

### Down-regulated genes involved in the response to cotton boll weevil feeding

Several putative genes involved in cell wall formation and degradation were identified as down-regulated. Contig12858 was one of the most highly down-regulated genes in our study (log2-fold change = −34.07, Additional file 2). Contig12858 is similar to genes belonging to the invertase/pectin methylesterase inhibitor family, from which some genes are known to be involved in cell wall modification but have also been reported to play an important role in basal disease resistance [47, 48]. Other genes with potential roles in cell wall reorganisation were down-regulated, such as an endoxyloglucan transferase (contig9845), which has a role in primary cell wall restructuring, as well as an alpha/beta hydrolase (contig12015) and a beta-expansin (contig9652) (Additional file 2). Interestingly, many down-regulated genes encoding heat shock proteins (Hsp) and one heat shock transcription factor (HSF) were observed. Among them, five belong to the small *Hsp* family, four to the *Hsp70* family and six to the *Hsp90* family (Additional file 2).

### Validation of transcriptome data and evaluation of the spatial expression pattern of defence-related genes in response to infestation by cotton boll weevil larvae using “*in situ*” qPCR

To better characterise the potential role of some genes in plant defence against insect herbivory, we selected 32 DEGs that responded to feeding by the cotton boll weevil larvae. These genes function in different levels of the biotic stress defence response signalling pathway (Figure 2) and were annotated in biological processes such as the response to chitin and/or cell death (Table 3). Initially, these were analysed with real-time qPCR on 6 mm cotton flower buds infected by larvae or the control samples for further validation of the transcriptome data. The expression patterns observed between the RNA sequencing (RNAseq) and qPCR methods were highly similar (Additional file 8), indicating the accuracy of our transcriptome profile. To verify this observation, we calculated the Pearson correlation coefficient (r) between the different methods for all transcripts, and a correlation coefficient of 0.9406 (P ≤ 0.05) was observed.

**Figure 4:**
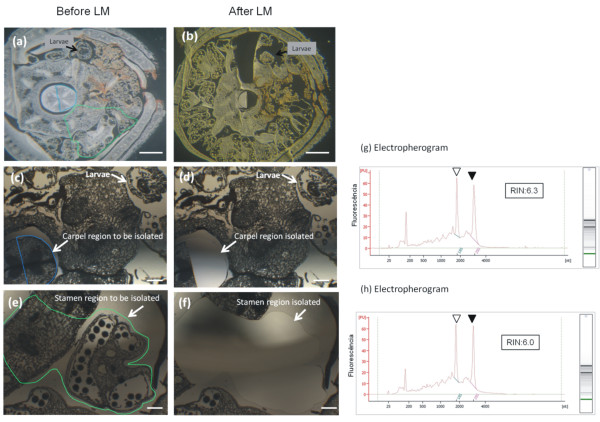
**Isolation of cotton tissues from paraffin-embedded sections by laser microdissection (LMD).** Isolation of cotton tissues from paraffin-embedded sections by laser microdissection (LMD). Sections before LMD **(a, c, e)**, and sections after LM **(b, d, f)**. The area selected for laser microdissection is outlined in green (a region near the damage caused by larvae feeding, which comprised the stamen tissue, viewed at **a**, **c** and **d**) or blue (a region farther from the injured area, which comprised the carpel tissue, viewed at **a**, **e**, and **f**). The assessment of extracted RNA integrity from the stamen and carpel are shown in **g** and **h**, respectively. Electropherograms were obtained with an Agilent 2100 Bioanalyser. Open and closed arrowheads indicate the 18S and 28S ribosomal RNA peaks, respectively. RNA quality is expressed as the RNA integrity number (RIN). Scale bars = 100 μm.

**Figure 5:**
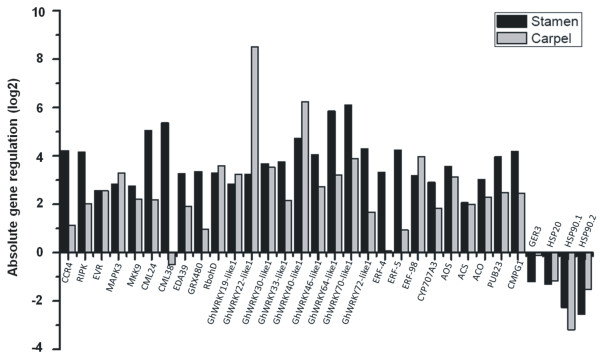
**Comparison of expression levels by “** ***in situ*** **” qPCR of a subset of 32 DEG.** These genes were examined from two different areas (stamen and carpel) isolated from 6 mm cotton flower buds infested by cotton boll weevil larvae by laser microdissection (LMD) in relation to the control. The reference genes *GhACT4* and *GhFBX6* were used to normalise the qPCR data. The relative expression level was calculated using the relative expression software tool (REST^©^), and a subsequent statistical test of the analysed C_P_ values by a *Pair-Wise Fixed Reallocation Randomization Test* was performed.

To evaluate the spatial expression pattern of these genes by “*in situ*” qPCR, two different areas of the 6 mm cotton flower buds were isolated by laser microdissection (LMD): the stamens (near the feeding site) and the carpels (away from the injured area) (Figure 4). Among the 28 up-regulated genes selected for analysis, 27 were up-regulated in both organs. Only *CML38* showed a contrasting expression pattern of up-regulation in the stamens (near the feeding site) but down-regulation in the carpels (away from the feeding site) (Figure 5). The spatial expression analysis of four down-regulated genes showed that all four were repressed in stamens and carpels (Figure 5). We also evaluated if the expression near the feeding site (stamens) was significantly different from the expression away from the damaged tissue (carpels). Among the 32 genes tested by qPCR, transcripts from 24 genes were more highly expressed near the feeding site (Figure 5). A few genes, such as the transcription factors *GhWRKY19-like1*, *GhWRKY22-like1*, *GhWRKY40-like1* and *ERF-98* and the *RbohH* and *HSP90.1* genes were more highly expressed in carpel tissues compared to stamen tissues (Figure 5 and Additional file 9).

### The differentially expressed transcriptome of cotton in response to boll weevil larvae feeding

**Figure 6:**
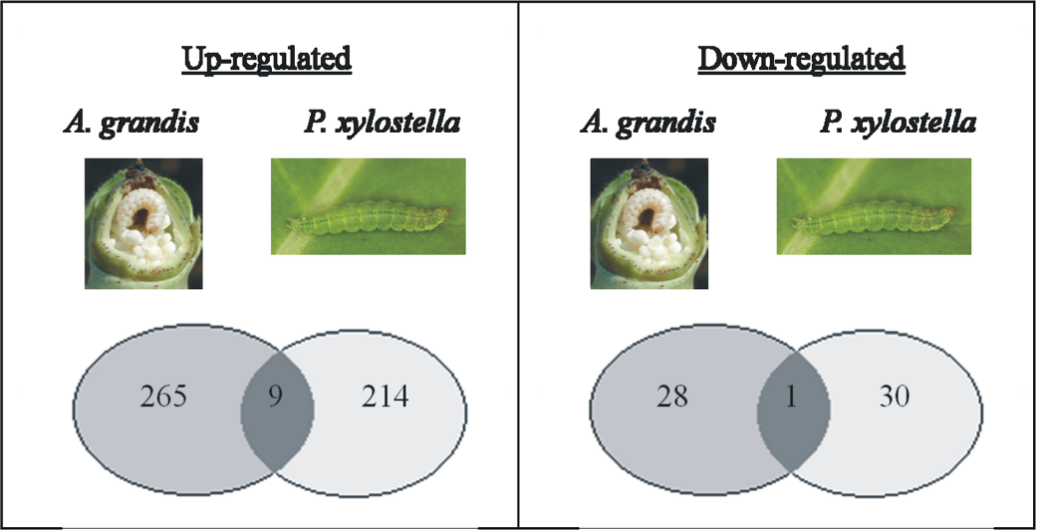
**Venn diagram comparing the cotton boll weevil (** ***Anthonomus grandis*** **) induced transcriptome with the response to another tissue-chewing pest,** ***Plutella xylostella*** **.** The number of induced (up-regulated, left column) and repressed (down-regulated, right column) genes after 48 h of cotton boll weevil feeding (*A. grandis*) was compared to the response of another 24 h herbivory-treatment previously published [39]. Selected genes have a p-value ≤ 0.05 and |logFC| ≥ 2.0.

To further evaluate whether cotton and *Arabidopsis* share a common gene set in response to tissue-chewing pests, we compared the differentially expressed transcriptome with the publicly available microarray data set GEO: GSE10681 (*Arabidopsis* leaf transcriptome in response to diamond back moth (DBM, *Plutella xylostella* larvae feeding). We also used a GSEA approach to determine the genes and pathways that were relevant in response to 24 hours of continuous DBM feeding and identified the biological processes that were overrepresented in the ordered list of DEG in this experiment (data not shown). We found several similarities between the cotton transcriptome and the public domain microarray data in terms of enriched biological processes. Biological processes related to hormone biosynthesis and signalling, such as JA, SA, ET and ABA, the responses to organic substances, the negative regulation of programmed cell death, the detection of biotic stimuli, systemic acquired resistance and the regulation of the immune response were overrepresented in both GSEA analyses. However, there were obvious differences in the enriched biological processes, including the response to auxin, gibberellin stimulus and secondary metabolite biosynthetic processes. These biological processes are overrepresented only in the leaf transcriptome analysis of *Arabidopsis* infested by DMB. On the other hand, the response to chitin and signalling are biological processes overrepresented in cotton flower buds damaged by cotton boll weevil larvae. There were only nine up-regulated genes that overlapped between the cotton larvae in floral tissue and the leaf injured by diamond back moth (Figure 6). Among them, there were transcripts encoding proteins that repress JA signalling, such as JAZ1 (At1g19180), JAZ7 (At3g34600), and JAS1 (At5g13220) as well as a disease resistance protein LRR (At2g34930) that specifically recognises pathogen effectors, resulting in effector-triggered immunity (ETI). Another gene represented in both data sets is a lipoxygenase (LoX4, At1g72520) that dioxygenates unsaturated fatty acids, leading to the lipoperoxidation of biological membranes. LoXs are involved in the apoptosis (programmed cell death) pathway and in biotic and abiotic stress responses in plants [49]. Finally, the gene 2-oxoglutarate and Fe(II)-dependent oxygenase (At5g05600), which is involved in the phenylpropanoid pathway and implicated in the synthesis of defence compounds, was found in both data sets and was also induced by *Pieris brassicae* eggs and *Pieris rapae* herbivory [17]. We found only one gene that was down-regulated in both experiments: a germin-like protein (GER3) with oxalate oxidase activity leading to hydrogen peroxide (H_2_O_2_) production, which is a ROS that is implicated in the herbivory-induced response in plants [17].

## Discussion

*G. hirsutum* L. is an allotetraploid species with a large and complex genome, and comprehensive sequence information describing the genome is still incomplete. Moreover, previous studies have provided limited information on the transcriptional dynamics during the cotton defence response [50]. The recent availability of high-throughput sequencing technologies provides an unprecedented opportunity to thoroughly explore the defence response using large-scale expression profile analysis despite uncharacterised genomic sequences. In the present study, using massive parallel mRNAseq, we report the transcriptional changes in *G. hirsutum* L. flower buds in response to cotton boll weevil (*A. grandis*) larvae feeding. To this end, eggs from *A. grandis* that were ready to hatch were placed on stamens inside 6 mm flower buds, the stage at which the round unicellular microspores are found in the locules. Cotton boll weevil larvae chew for ~48 h on flower tissues, representing the early steps of insect damage. It has been suggested that pollen grains are the initial target of the larvae after egg hatching [51]. Subsequently, the larvae migrate to the ovary where they feed in the ovules and conclude their life cycle, leading to the abscission of floral structures [51].

In this study, we generated over 327 million sequence reads (100 bp in length) of raw sequence data using the Illumina sequencing of two biological replicates from 6 mm cotton flower buds damaged by cotton boll weevil larvae and undamaged flower buds. At the current level of sampling (at least 74 million tags per sample), the sensitivity of the RNASeq approach allowed the accurate identification of differentially expressed genes.

A previous study in cotton showed by RNA-Seq that the transcriptome changes after pathogen (*Verticillium dahliae*) inoculation, although they generated only 27 million tags of 21 bp in length [50]. Dubey et al. [4] analysed the leaf transcriptome of cotton plants infested by aphids and whiteflies, generating a total of 3.8 million high quality reads through RNA-seq. In our study, the number of up-regulated genes (402) was much greater than the number of down-regulated genes (41) after damage by cotton boll weevil larvae. These results are supported by previous reports showing that larval feeding stimulates induction rather than suppression of genes [18, 39]. Several studies have examined the transcriptional profiles of different modalities of attack, such as pathogen or insect [15, 18, 31]. However, our results contrast with results observed in cotton plants damaged by whiteflies and aphids, in which the number of down-regulated genes was greater than the number of up-regulated genes in both cases after damage [4].

The MapMan analysis of the DEGs generated a representative overview of the plant response after larvae damage (Figure 2). This analysis suggested that the cotton plant could perceive boll weevil larvae-derived physical and chemical cues such as compounds in the oviposition fluid, chitin, and insect oral secretions. These elicitors dramatically alter the expression of several genes in the plant immune response pathways that are involved in biotic stress response. The herbivory-induced changes are mediated by elaborate signalling networks, including receptors/sensors, Ca2+ influx, kinase cascades, transcription factors, reactive oxygen species and phytohormone signalling pathways. We were able to identify genes belonging to all major networks previously described and thus relate these to the plant response to herbivory.

The plant immune system is activated by various herbivore (or microbial)-associated molecular patterns (HAMP or MAMP) that are detected as non-self molecules. Such patterns are recognised by immune receptors that are either cytoplasmic or localised on the plasma membrane. Cell surface receptors include receptor-like kinases (RLK) that frequently contain extracellular leucine-rich repeats and an intracellular kinase domain for the activation of downstream signalling, as well as receptor-like proteins (RLP) that lack this signalling domain. It is therefore hypothesised that RLKs are required for RLPs to activate downstream signalling [52]. Several putative receptor-like kinases, such as the homologues of *CCR4, RIPK* and *EVR*, were up-regulated after cotton boll weevil larvae damage (Table 3). *CCR4* encodes a transmembrane protein kinase (RLK) previously characterised as a key gene in the plant-insect interaction. Little et al. [17] showed that 41 RLKs, including *CCR4*, were induced by oviposition by *P. brassicae,* suggesting that RLKs play an important role in the detection of herbivore-associated molecular patterns. Interestingly, *CCR4* was significantly more highly expressed in the stamen close to the larval damage than in the carpel, as assessed by our *in situ* qPCR analysis (Figure 5). Furthermore, the *RIPK* gene encodes a cytoplasmic NB-LRR immune receptor that recognises the AvrB and AvrRpm1 effectors from *Pseudomonas syringae*, leading to the activation of ETI [53]. This gene was also more highly expressed in the stamen than in the carpel tissue in our *in situ* qPCR analysis. Another leucine rich repeat transmembrane protein kinases analysed in our study, *EVR*, was equally induced by boll weevil larvae in the stamen and the carpel, is also expressed in response to *P. syringae,* and is involved in the regulation of cell death and innate immunity (Figure 5) [52].

The recognition of conserved HAMP, such as chitin or fatty acid-amino acid conjugates (FAC, the major component of insect oral secretions) by PRR triggers intracellular signalling via a mitogen-activated protein kinase (*MAPK*, *MAPKK*, *MAPKKK*) cascade [27]. Our study identified homologues to *MAPK3* (At3g45640) and *MKK9* (At1g73500) genes with the GO term “response to chitin”. The *in situ* qPCR analysis showed that these genes were highly expressed in the stamen and carpel from cotton flower buds infested by boll weevil larvae (Figure 5). *MAPK*, *MAPKK*, and *MAPKKK* activate several transcription factors such as ERFs and WRKYs during the response to pest attacks, as has been shown for *ERF-5* [54]. A previous study in *Arabidopsis* showed that *ERF-5* is phosphorylated by *MAPK3* (At3g45640), suggesting that *ERF-5* might play an important role in plant defence by positively regulating ethylene (ET) signalling [54]. The *GhERF-4* and *GhERF-5* genes were more highly expressed in the stamen than in the carpel, and the *GhERF-98* gene was slightly more highly expressed in the carpel (Figure 5). *ERF-4*, *ERF-5* and *ERF-98* belong to the AP2/EREB family and were previously identified in the response to nematode or fungal inoculation (*Alternaria brassicicola*) as well as in response to chitin, a main elicitor of the plant defence response against insects [31, 55].

RNAseq analysis identified transcripts for 88 TFs that were differentially expressed in 6 mm cotton flower buds damaged by boll weevil larvae. In the present study, we selected the 21 cotton genes identified as WRKY for a phylogenetic analysis in combination with the full set of *Arabidopsis* WRKY genes. The expression patterns of nine *WRKY* genes were explored in detail with a combination of LMD and qPCR. The transcript level of *GhWRKY30-like1* gene was the same in stamen and carpel tissue and was induced relative to the control. *GhWRKY33-like, GhWRKY46-like1, GhWRKY64-like1, GhWRKY70-like1* and *GhWRKY72-like1* were more highly expressed near the damage in stamen tissues. In *Arabidopsis*, it was also shown that *WRKY46* is specifically induced by salicylic acid (SA) and infection by the biotrophic pathogen *P. syringae*, working redundantly with the structurally related genes *WRKY70* and *WRKY53* to positively regulate basal resistance to *P. syringae* [56]. Previous results have shown that the *Arabidopsis WRKY* genes are rapidly and strongly induced by chitin [31]. Some *WRKY*s are target genes of the upstream MAPK3 (At3g45640), such as *WRKY33*. Studies in *Arabidopsis* have shown that in response to pathogen invasion, *MAPK3* (At3g45640) phosphorylates *WRKY33*, which directly binds to the W-boxes in the promoter of the *ACS6* gene *in vivo*, suggesting that *WRKY33* is directly involved in the activation of *ACS6* expression downstream of the *MAPK3* cascade [57]. This signalling event produces high levels of ethylene, which plays an important role in plant immunity. Moreover, *AtWRKY33* is required to activate the synthesis of antimicrobial substances such as camalexin [34]. Interestingly, the other three *WRKY* genes (*GhWRKY19-like1, GhWRKY22-like1 and GhWRKY40-like1*) showed a higher expression in the carpel than in the stamen. In an elegant series of experiments, Asai et al. [58] showed that *AtWRKY22* functions downstream of the flagellin receptor, a leucine-rich repeat (LRR) receptor kinase, and that a mitogen-activated protein kinase (MAPK) cascade conferred resistance to bacterial (*P. syringae*) and fungal (*Botrytis cinerea*) pathogens. These results strongly indicate that signalling events initiated by boll weevil larvae feeding might converge on a conserved system of pathogen-associated molecular pattern recognition, a MAPK cascade and transcription factors.

Relatively little is known about the signal transduction pathways triggered by insect damage. Calcium ions (Ca^2+^) have been implicated as a second messenger in many plant signalling pathways including responses to herbivory [12]. It has been shown that mechanical wounding and insect damage trigger a transient increase in cytosolic Ca^2+^ levels [59]. For instance, the Egyptian cotton worm (*Spodoptera littoralis)* caused a transient increase in cytosolic Ca^2+^ in *Phaseolus lunatus* cells adjacent to the insect damage [59]. Cotton genes encoding calmodulin-like proteins (*EDA39*, *CML38* and *CML24)* showed a higher expression close to the damage (stamen tissues) than in carpels, suggesting that these proteins might be a component of Ca^2+^ signalling that modulate plant defence responses against herbivory attack.

Several studies have revealed that ROS are implicated in herbivory-induced responses in plants [12, 43, 60]. Superoxide anion (O^2−^), hydrogen peroxide (H_2_O_2_), singlet oxygen (^1^O_2_), and hydroxyl radical (**·**OH) are collectively called ROS; they are produced in mitochondria, chloroplasts, and peroxisomes, as well as on the external surfaces of plasma membranes. Feeding of *Helicoverpa zea* on soybean, as well as *S. littoralis* and *Tetranychus urticae* feeding on *Medicago truncatula* plants, increase ROS levels [12, 61]. Our analysis identified several transcripts encoding enzymes directly involved in oxidative stress that are up-regulated in response to feeding by boll weevil larvae. *In situ* qPCR analysis showed that transcripts encoding an NADPH oxidase (*RbohD*) were expressed at a higher level in carpel tissues than in stamen tissues compared to the control samples (Figure 5). *AtrbohD* was largely responsible for the accumulation of ROS during the defence response in *Arabidopsis* against *P. syringae* and *Peronospora parasitica* [60]. Our results suggest that the oxidative burst is also part of the cotton plant’s response to boll weevil larvae feeding.

The boll weevil larval damage in flower buds also induced the expression of genes associated with phytohormone biosynthetic processes and signalling. Among the gene transcripts associated with JA, ET and ABA biosynthesis selected for analysis by qPCR were *AOS*, *ACS*, *ACO* and *CYP701A3*. All of these genes showed a significantly higher expression in the stamen and carpel tissue compared to the control samples. Among the JA metabolism genes that modulate plant responses to biotic stress by boll weevil larvae are *AOS*, which encodes the second enzyme involved in JA biosynthesis, and *JMT*, which is involved in the synthesis of the volatile compound methyl-JA (MeJA) used for plant defence. Jasmonate plays a central role in regulating the defence responses to herbivores that inflict various types of tissue damage. Jasmonate mutants are affected in their resistance to a wide range of arthropod herbivores, including caterpillars (Lepidoptera), beetles (Coleoptera), thrips (Thysanoptera) and leafhoppers (Homoptera) [9, 59, 62]. Jasmonate has been associated with the synthesis of proteinase inhibitors (PI), defence-related volatile compounds and secondary metabolites, such as nicotine, active phenolics and phytoalexins [12]. The genes involved in the ET biosynthesis pathway, *ACS* and *ACO*, were also up-regulated by boll weevil larvae damage. The primary function of ET in plant resistance to herbivores is the fine-tuning of JA-induced responses [12]. In tomato plants, ET potentiates the JA-induced transcript accumulation of secondary metabolites such as PIs [63]. The treatment of *Arabidopsis* plants with ethephon, a synthetic ET donor, transiently elevates the levels of JA and *AOS* transcripts [64]. Blocking the perception of ET with 1-MCP diminishes herbivory-induced volatile emission, which is mainly regulated by JA [65]. By using plants that ectopically express a loss-of-function ETR gene, von Dahl et al. [66] showed that ET signalling is crucial for increasing basal levels of nicotine, an effective defence against herbivory. These results indicated that JA and ET may be involved in elevating the basal resistance of cotton plants to herbivory.

The ubiquitin-ligase genes are also known to play a role in the plant defence response [67]. Yang et al. [68] showed that the E3 ligase activity of *Arabidopsis* PUB17 is required for the initiation of the hypersensitive response (HR). Therefore, two predicted members of this family, *PUB23* and *CMPG1*, which are among the DEGs in our experiment and were annotated in response to chitin and/or death, were also analysed by *in situ* qPCR. Spatial analysis of gene expression revealed that the transcripts of both genes were strongly up-regulated at the site close to the damage (stamen) compared to carpel tissues. These genes are among the 30 ubiquitin-ligase genes that up-regulated either 15 or 30 min after chitooctaose treatment in *Arabidopsis* [31]. Previous studies have shown that the expression of the *PUB23* gene in *Arabidopsi*s was induced by PAMPs and infection by pathogens. Moreover, the *pub22/pub23/pub24* triple mutant displayed negative regulation of PAMP-triggered immunity [69].

Among the repressed gene transcripts related to the herbivory response, we identified transcripts for one germin-like protein (GER3) and many genes encoding heat shock proteins (Hsp). qPCR analysis revealed that the *GER3* transcript was strongly down-regulated in the stamen and in the carpel (Figure 5). Interestingly, *GER3* is included in the overlap between the down-regulated genes of the leaf transcriptome analysis of *Arabidopsis* damaged by DMB larvae and our study (Figure 6). GER3 leads to hydrogen peroxide (H_2_O_2_) production, a type of ROS that is implicated in the herbivory-induced response in plants, and was also repressed by *P. brassicae* eggs and *P. rapae* herbivory in a study in *A. thaliana* [17]. The transcriptional repression of genes encoding the heat shock proteins *HSP20*, *HSP90.1* and *HSP90.2* was observed in both stamen and carpel tissues (Figure 5). In many plant species, HSP90 isoforms are required for disease resistance against invading pathogens. HSP90 interacts with the disease resistance signalling components SGT1b and RAR1 and is required for RPS2-mediated resistance, a disease resistance (R) intracellular protein that specifically recognises pathogen effectors. For example, the *AtHSP90.1* and *AtHSP90.2* genes in *Arabidopsis* are required for RPS2-mediated resistance against *P. syringae* expressing *AvrRpt2* and for *RPM1*-mediated resistance to *P. syringae* expressing *AvrRPM1*, respectively [70–72]. HSP90 is also essential for Rx-mediated resistance to Potato virus X (PVX), N-mediated resistance to Tobacco mosaic virus, and Pto-mediated resistance to *P. syringae* expressing *AvrPto* [73, 74]. In contrast, the *hsp90.2-3* mutant with a point mutation in the ATP-binding domain of *AtHSP90.2* is known to be more sensitive to biotrophic pathogens. In our results, several *hsp* genes were down-regulated, suggesting that larvae attempt to overcome the plant immune defence triggered by the effector.

## Conclusion

Alternative strategies for protecting crops from insect pests are exploring the endogenous resistance mechanisms exhibited by plants to most herbivore insects through a greater understanding of induced defences in plants. This study aimed to unravel the changes in the transcriptome of *G. hirsutum* flower buds in response to feeding by cotton boll weevil (*A. grandis*) larvae. A large number of cotton transcripts and biological processes were significantly altered upon larvae infestation. Among the changes observed, we highlighted the induction of transcripts for the receptors/sensors that recognise chitin, insect oral secretions and signals from injured plant cells; differential modulation of transcripts encoding enzymes related to kinase cascades; transcription factors (such as WRKY and ERF); Ca^2+^ influx; reactive oxygen species; and modulation of transcripts encoding enzymes from phytohormone signalling pathways, mainly the JA and ET pathways. Cotton boll weevil larvae feeding affected many genes that have also been shown to be regulated in response to microbial or fungal infection, indicating the existence of complex crosstalk in the response to these different pathogens. The qPCR analysis associated with microdissection showed that almost all of the genes were more highly expressed in the stamen, the region close to the damage caused by larvae feeding, than in the carpel, farther away from the injured area. However, herbivory-induced defence responses in cotton flower buds were observed not only in the wounded regions but also in undamaged regions of the damaged flower buds. It is possible that a signal travels to other parts of the flower bud that transmit an herbivory alert. Although the identity of the mobile signal responsible for this systemic response remains unknown in many plants, studies performed in solanaceous plants demonstrated that herbivory rapidly induces a short-distance mobile signal that travels and activates MAPKs and triggers JA accumulation. Deciphering the signals that regulate herbivore-responsive gene expression in *G. hirsutum* flower buds might provide new strategies to manipulate this response for the production of insect-resistant transgenic plants.

## Methods

### Plant material and *A. grandis*infestation assay

Cotton (*Gossypium hirsutum* L. variety “BRS Cedro”) plants were grown under a controlled temperature (27 ± 2°C) and natural photoperiod at Embrapa Genetic Resources and Biotechnology in Brasilia (DF, Brazil). Three-month old plants containing 6 mm flower buds were selected for the experiments. A population of *A. grandis* (Coleoptera: Curculionidae) was maintained at 27 ± 2°C, 70 ± 10% relative humidity, and a photoperiod of 14 h. Insects were routinely maintained on a standard rearing diet [75]. A 6 mm cotton flower bud previously drilled with a needle was inoculated with an *A. grandis* egg containing an active embryo. The orifice resulting from drilling was sealed with Vaseline to prevent dehydration of the egg. The hatching of larvae occurred approximately 2 h after egg inoculation. The larvae were removed with tweezers from the flower buds 48 h after inoculation, and four flower buds from different plants were immediately frozen in liquid nitrogen for the isolation of total RNA to perform RNAseq experiments. Similarly, a set of twelve flower buds infected with larvae were collected and fixed in cold ethanol:acetic acid (3:1) to subsequently isolate the selected tissue through laser microdissection. The insect infection experiments were performed in two biological replicates. The control samples were drilled cotton flower buds into which no eggs were introduced.

### RNA isolation and sample preparation

Total RNA extraction was performed from 100 mg of macerated plant tissue in liquid nitrogen using the Invisorb Spin Plant RNA Mini kit (Invitek) according to the manufacturer’s protocol. RNA quality and quantity were determined using a Nanodrop 2000 (Thermo Scientific) and a Bioanalyser Chip RNA 7500 series II (Agilent), respectively. The analysis of RNA integrity was judged by the RNA Integrity Number (RIN), which was calculated with 2100 Expert Software. The software algorithm, which was developed by Schroeder et al. [76], categorises total RNA quality on a scale from 1 to 10, in which 10 corresponds to intact RNA and 1 corresponds to highly degraded RNA. Generally, plant RNA with a RIN value of 6–7 was of acceptable quality for qPCR and RNAseq analyses. RNA Integrity Number (RIN) values were greater than 9.0 for all samples. Two biological replicates of inoculated flower buds and controls were used for transcriptome sequencing. Library preparation and massive parallel sequencing were performed by Eurofins MWG Operon (Huntsville, AL). Sequencing libraries were prepared using NEBNext Ultra Directional RNA Library Prep Kit (New England Biolabs, MA), which included Poly-A-containing mRNA isolation from 5 μg total RNA using two rounds of purification with poly-T oligo-attached magnetic beads and fragmentation by sonication. First strand cDNA was generated using reverse transcriptase and random primers. Following the second strand cDNA synthesis and adaptor ligation, 400 bp cDNA fragments were isolated using gel electrophoresis and amplified by PCR. The products were loaded onto an Illumina HiSeq™ 2000 instrument and subjected to 200 cycles of paired-end (2 × 100 bp) sequencing. The processing of fluorescent images into sequences, base-calling, and quality value calculations were performed using the Illumina data processing pipeline (version 1.8).

### Mapping of short reads and assessment of differential gene expression

Raw reads were filtered to obtain high-quality reads by removing low-quality reads containing more than 30% bases with Q < 20. After trimming low-quality bases from the 5′ and 3′ ends of the remaining reads, the resulting high-quality reads were aligned against a *G. hirsutum* expressed sequence tag (EST) assembly (with 28,432 unique genes/contigs) as the transcriptome reference (cotton EST database; http://www.leonxie.com) [77] using the BWA package [78]. Differential expression was estimated and tested with the DEGseq software package in R-bioconductor 2.15 for each library with reference to the control [79]. The count data were normalised to the total number of counts, accounting for the variance and the mean of the biological replicates. Contigs with adjusted p-value ≤ 0.05 and estimated absolute log_2_ (FC) ≥ 2 were determined to be significantly differentially expressed genes (DEG) and were selected.

### Functional annotation

The DEG were subjected to blast using the program Blastx with the TAIR 9 protein database and further classified into categories according to the GO classification system (Additional file 2). The Blast2GO program (http://www.blast2go.com/b2ghome) [80] was also used to confirm the annotation of transcripts (Additional file 2). RNA-seq data can be accessed at the NCBI bioprojects under the accession number PRJNA245406.

To identify relevant molecular mechanisms potentially associated with the response to cotton boll weevil larvae development within cotton flower buds, GSEA (Gene Set Enrichment Analysis) was performed [81]. A gene set was defined as all DEG, with annotations according to *A. thaliana*, that share the same ontology based on the GO database. The GSEA method identified biological processes (BP), molecular functions (MF) and cellular components (CC) that were overrepresented among the list of DEG (Figure 1 and Additional file 3). The overrepresentation was assessed with a statistical score based on a hypergeometric test with p-values ≤ 0.005.

In addition, the differentially expressed genes were also functionally analysed using MapMan software, which is a user-driven tool that displays large genomic datasets onto diagrams of metabolic pathways or other processes such as biotic stress [82] (Figure 2, adapted by [10]). Finally, the differentially expressed genes were compared with the public databases generated from microarray analysis of *Arabidopsis thaliana* leaves that were infested by diamond back moth (*Plutella xylostella*) larvae 24 h after the onset of herbivory (GEO:GSE10681) (Figure 6) [39].

### Defence-related genes in response to infestation by cotton boll weevil larvae used for qPCR analysis

Real time PCR (qPCR) assays were performed on 32 differentially expressed genes identified by RNAseq analysis (Additional file 9). Twenty-eight of them were reported as up-regulated in cotton boll weevil-infested flower buds: three receptor-like kinase proteins (*CCR4*, *RIPK* and *EVR*); two proteins from the mitogen-activated kinase signalling cascade (*MAPK3*, *MKK9*); three calmodulin-like proteins (*CML28*, *CML24*, *EDA39*); two genes from the oxidative burst process (*GRX480*, *RbohD*); twelve TFs, including nine WRKY transcription factors (*WRKY19, 22, 30, 33, 40, 46, 64, 70* and *72*) and three AP2/ERE transcription factors (*ERF-4*, *ERF-5*, *ERF-98*); four genes involved in phytohormone biosynthesis (*CPY707A3*, *AOS*, *ACS*, *ACO*); and two ubiquitin ligase proteins involved in targeted protein degradation (*PUB23* and *CMPG1*). Among the down-regulated DEGs, we evaluated the expression pattern of a germin-like protein (*GER3*) and three members of the heat shock protein family (*HSP90.1*, *HSP90.2* and *HSP20*). Primers were designed using *Primer 3* software tools (http://frodo.wi.mit.edu/primer3/) with a melting temperature of 60°C, an amplicon length of 150 to 200 bp, and a GC content of 50 to 60% [83].

### Tissue fixation, embedding, sectioning, and laser microdissection

To assess the spatial expression pattern of the genes described above, tissues from two different areas were isolated from 6 mm cotton flower buds infested by cotton boll weevil larvae using laser microdissection (LMD). These areas (Figure 4) included the following: (a) a region near the damage caused by larvae feeding, consisting of stamen tissues and (b) another region farther away from the injured area, consisting of carpel tissue. The 6 mm cotton flower buds infested by larvae and control samples were fixed overnight at 4°C in Farmer’s Fixative (3:1 ethanol:acetic acid) and dehydrated. After fixation, samples were prepared as previously described [84]. Briefly, the samples were subjected to an ethanol/xylene series followed by a xylene/paraplast series before being embedded in paraplast X-tra (Sigma-Aldrich). Embedded samples were sectioned using a rotatory microtome (HM 325, Microm). The 16-μm-thick sections from the infected flower buds and control samples were mounted on Leica FrameSlides PET-Membrane 1.4 μm slides with methanol (No. 11505151). The slides were dried at 40°C for 5 min and then stored at 4°C until laser microdissection was performed. Laser microdissection was performed using a Leica LDM CTR 6500.

### RNA preparation, RNA amplification, and “*in situ*” qPCR

Isolated areas of carpel and stamen with biological replicates of infested and control samples (total of 180–200 microdissected cuts for each sample) were collected in microfuge tubes containing 100 μl RNALater solution (Ambion) and were used for RNA isolation (Figure 4). RNA was extracted from the captured tissues using the RNeasy Plant Mini kit (Qiagen) according to the manufacturer’s protocol, including a 15 min DNase incubation step (RNase-free DNase Set, Qiagen). The eluted RNA was concentrated under a vacuum until the remaining volume was approximately 10 μl. The quality of total RNA extracted from LMD-collected tissues was assessed using an RNA 6000 Pico kit on the Agilent 2100 Bioanalyser (Agilent Technologies). RNA Integrity Number (RIN) values were greater than 6.0 for all samples. The isolated total RNA was amplified using a MessageAmp aRNA Kit (Ambion) following the manufacturer’s instructions. One round of amplifications was performed, and the resulting anti-sense RNA (aRNA) was quantified by OD_260_. Equal amounts of aRNA (5.0 μg) of the two samples in biological replicates were reverse transcribed using 0.5 μl of Random Primers (C1181, Promega) and Superscript III reverse transcriptase (Invitrogen) according to the manufacturer’s instructions. Polymerase chain reactions were performed in the 7500 Fast Real-Time PCR detection system (Applied Biosystem) and SYBR® Green was used to monitor dsDNA synthesis. PCR efficiencies and the optimal quantification cycle threshold or Cq values were estimated using the online Real time PCR *Miner* [85]. Two independent biological samples for each experimental condition were evaluated using triplet technical replicates. The reference genes *GhACT4* and *GhFBX6* were used to normalise the qPCR data and have been discussed previously [86] (Additional file 9). The relative expression level was calculated using the relative expression software tool (REST^©^), which compares two treatment groups with multiple data points in the sample compared to the control groups and calculates the relative expression ratio between them. The mathematical model used is based on the PCR efficiencies and the mean C_P_ deviation between the sample and control group of target genes and is normalised to the mean C_P_ deviation of the reference genes [83]. The advantage of REST is the use of a subsequent statistical test of the analysed C_P_ values by a *Pair-Wise Fixed Reallocation Randomization Test* [87].

### Phylogenetic analysis of WRKY genes involved in the response to cotton boll weevil feeding

To identify the twenty-one cotton WRKY genes that were differentially expressed in our RNAseq experiment, a phylogenetic tree based on the WRKY domain of *Arabidopsis* was conducted (Additional files 2 and 7). The WRKY domain boundary was defined as described in Eulgem et al. [33] (excluding the N-terminal domains of Group I). Motif detection was performed with MEME 4.0 software [88] and the multiple amino acid sequence alignment of WRKY domains were conducted using MUSCLE (http://www.ebi.ac.uk/Tools/msa/muscle/) from 72 AtWRKY protein sequences from *Arabidopsis* (downloaded from AtTFDB (http://arabidopsis.med.ohio-state.edu/AtTFDB/) and 21 cotton WRKY genes. Phylogenetic trees were constructed after using a model test to pick the most appropriate evolutionary model for our analysis in the Mega 5.2 program [89]. The best-fitting amino acid substitution model for these gene families was the JTT model [90] with an estimated γ-distribution parameter (G). The maximum-likelihood analyses were performed with the program PhyML version 3.0 (http://www.atgc-montpellier.fr/phyml/) [91] and assessment of node confidence was performed using 1,000 bootstrap replicates. The trees were visualised and optimised in the Mega 5.2 program (Figure 3).

## Electronic supplementary material

Additional file 1: **JPEG file showing contig size distribution from the aligned reads of transcriptome sequencing of cotton flower buds.** (TIFF 11 MB)

Additional file 2: **Full list of the up-regulated and down-regulated cotton genes found in the RNA-seq analysis.** Only those differentially expressed genes with significant expression changes (adjusted p-value ≤ 0.05, |logFC| ≥ 2.0) are shown. Columns A-G are the results from Blastx with *Arabidopsis*, and columns H-P are the results from the Blast2GO program. (XLS 780 KB)

Additional file 3: **Molecular function (A) and Cellular component (B) ontologies overrepresented by the Gene Set Enrichment Analysis (GSEA).** (TIFF 521 KB)

Additional file 4: **Subset of differentially expressed genes (DEG) in cotton flower buds in response to cotton boll weevil larvae feeding that were annotated with the GO term “plasma membrane”.** (XLS 44 KB)

Additional file 5: **Subset of differentially expressed genes (DEG) in cotton flower buds in response to cotton boll weevil larvae feeding that were annotated with the GO term “cell wall”.** (XLS 38 KB)

Additional file 6: **Biological processes (BP) overrepresented by the Gene Set Enrichment Analysis (GSEA).** (XLS 58 KB)

Additional file 7: **The twenty-one cotton WRKY genes that were differentially expressed in our RNAseq experiment, the domain sequence used in the phylogenetic tree, the protein sequence and the nucleotide sequence.** (XLS 77 KB)

Additional file 8: **Comparison of qPCR and RNA sequencing expression data.** Thirty-two differentially expressed genes responding to cotton boll weevil larvae feeding were analysed for RNA abundance using qPCR. The log fold-change in RNA sequencing was estimated and tested by DEGseq software, in which the count data were normalised to the total number of counts, taking the variance and the mean of the biological replicates into account for each library with reference to the control (blue bars). qPCR results (red bars) were calculated with the relative REST software using a mathematical model based on the PCR efficiencies and the mean C_P_ deviation between the sample and control group of target genes and were normalised to the mean C_P_ deviation of reference genes. We calculated the Pearson correlation coefficient (r) between the different methods for all transcripts. The correlation coefficient was 0.9406. (TIFF 8 MB)

Additional file 9: **Primers used for the qPCR expression analysis of DEG in tissues isolated from cotton flower buds infested by cotton boll weevil larvae.** (XLS 52 KB)
