# Supplementary figures and images for: Transcriptome analysis of Gossypium hirsutum flower buds infested by cotton boll weevil (Anthonomus grandis) larvae
Source: BMC Genomics. 2014 Oct 4;15(1):854. doi: 10.1186/1471-2164-15-854 (PMC4234063; doi:10.1186/1471-2164-15-854)

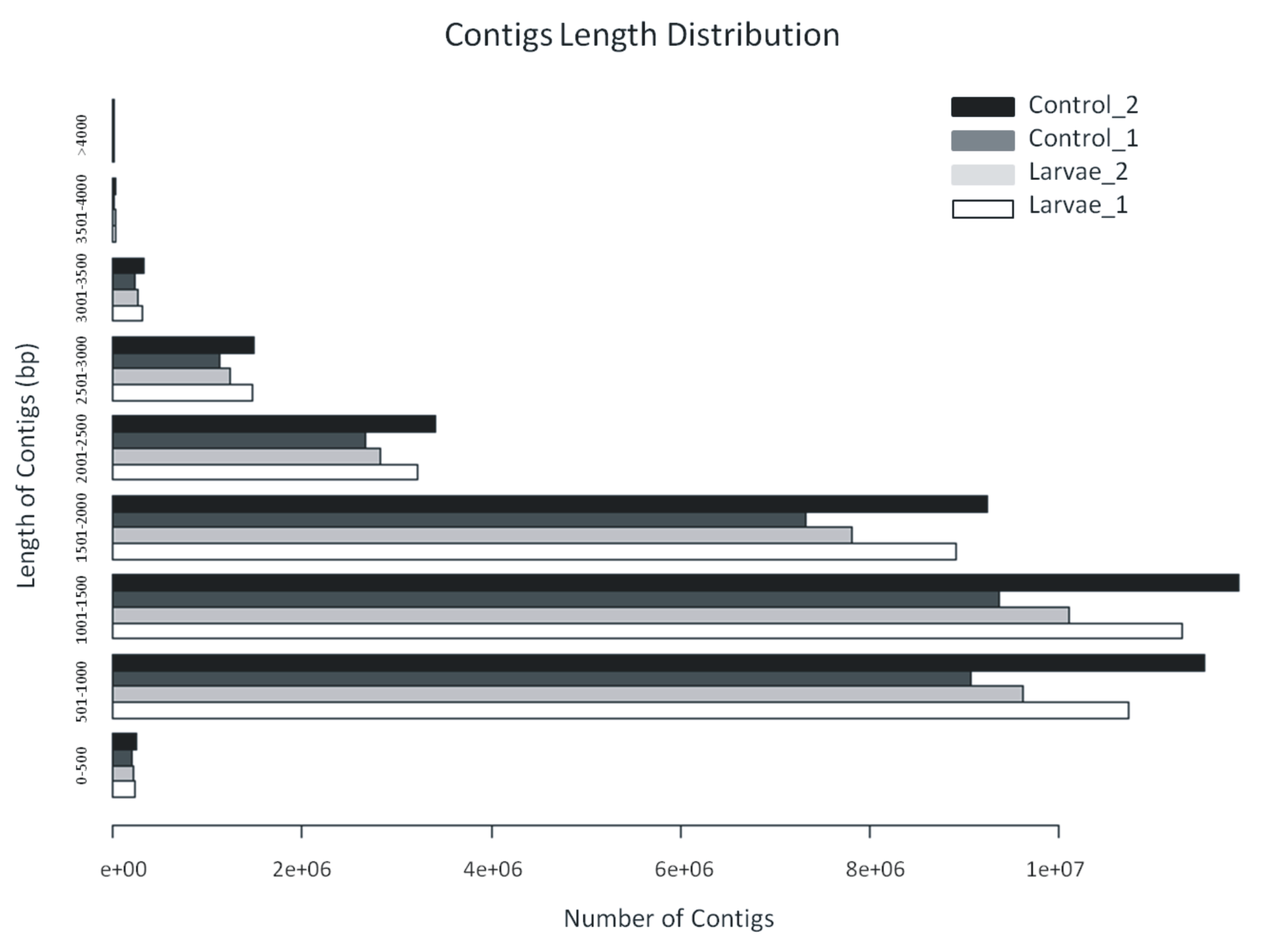

Supplement: Supplementary file 1 — Additional file 1: JPEG file showing contig size distribution from the aligned reads of transcriptome sequencing of cotton flower buds. (TIFF 11 MB) [file 12864_2014_6553_MOESM1_ESM.tiff]

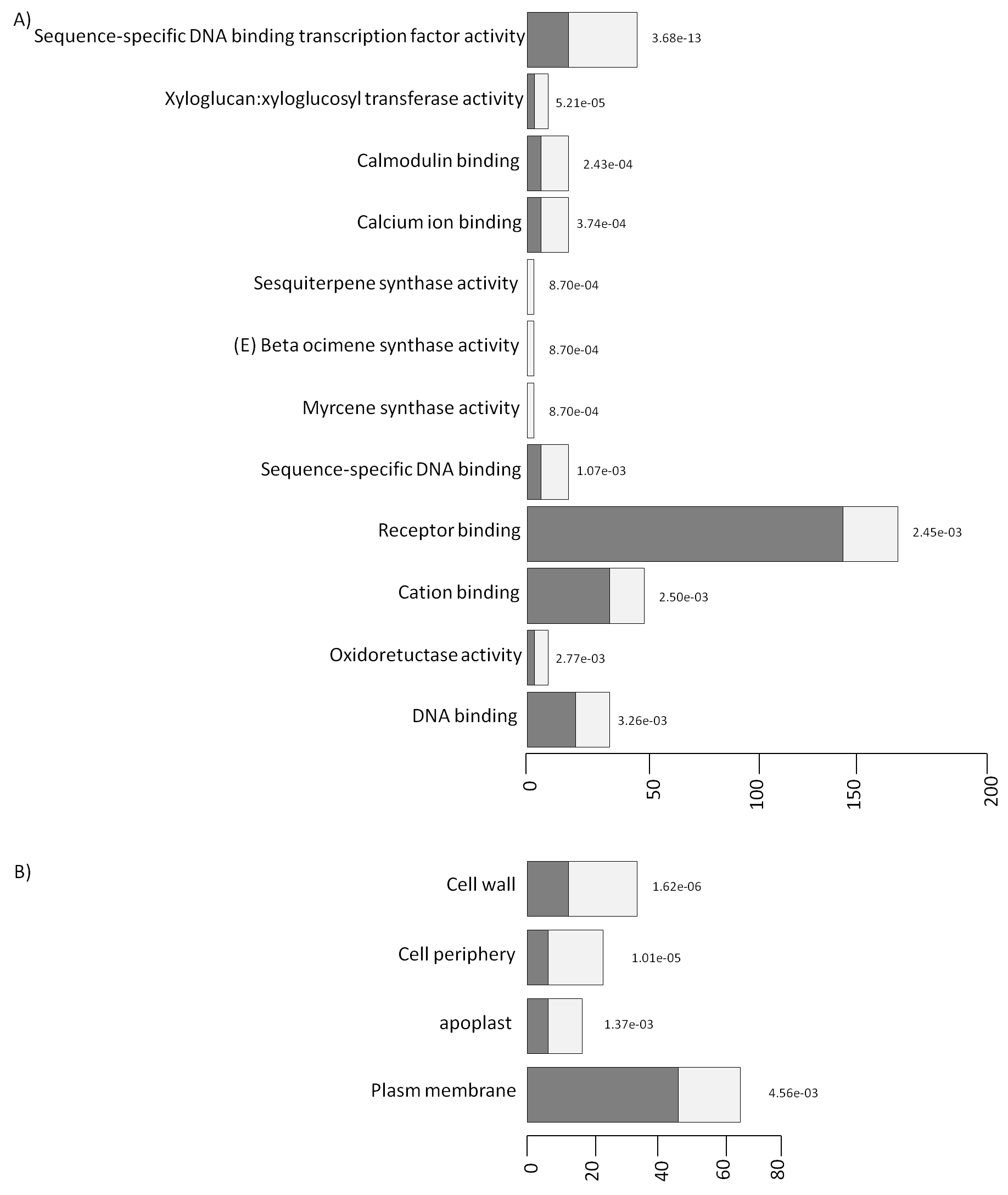

Supplement: Supplementary file 3 — Additional file 3: Molecular function (A) and Cellular component (B) ontologies overrepresented by the Gene Set Enrichment Analysis (GSEA). (TIFF 521 KB) [file 12864_2014_6553_MOESM3_ESM.tiff]

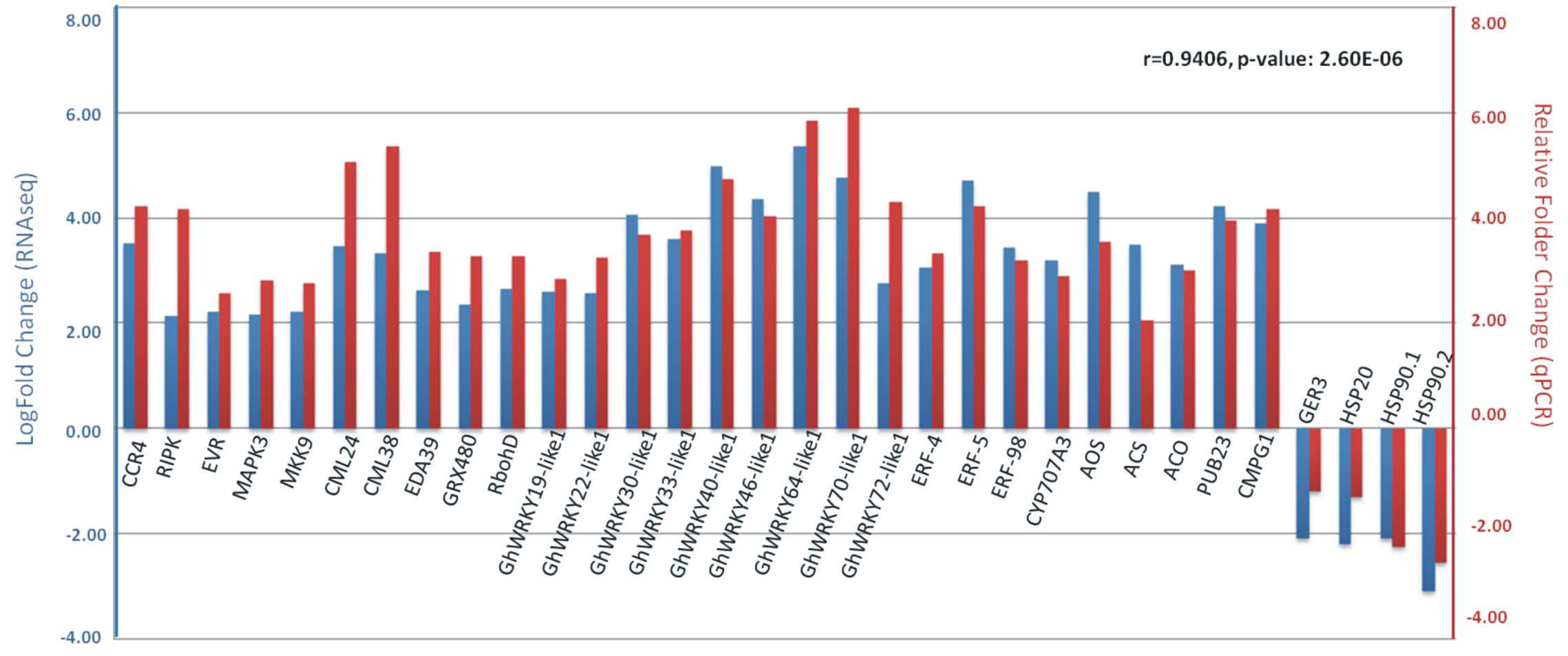

Supplement: Supplementary file 8 — Additional file 8: Comparison of qPCR and RNA sequencing expression data. Thirty-two differentially expressed genes responding to cotton boll weevil larvae feeding were analysed for RNA abundance using qPCR. The log fold-change in RNA sequencing was estimated and tested by DEGseq software, in which the count data were normalised to the total number of counts, taking the variance and the mean of the biological replicates into account for each library with reference to the control (blue bars). qPCR results (red bars) were calculated with the relative REST software using a mathematical model based on the PCR efficiencies and the mean CP deviation between the sample and control group of target genes and were normalised to the mean CP deviation of reference genes. We calculated the Pearson correlation coefficient (r) between the different methods for all transcripts. The correlation coefficient was 0.9406. (TIFF 8 MB) [file 12864_2014_6553_MOESM8_ESM.tiff]
